# Using Wearable Activity Trackers to Predict Type 2 Diabetes: Machine Learning–Based Cross-sectional Study of the UK Biobank Accelerometer Cohort

**DOI:** 10.2196/23364

**Published:** 2021-03-19

**Authors:** Benjamin Lam, Michael Catt, Sophie Cassidy, Jaume Bacardit, Philip Darke, Sam Butterfield, Ossama Alshabrawy, Michael Trenell, Paolo Missier

**Affiliations:** 1 School of Computing Newcastle University Newcastle upon Tyne United Kingdom; 2 Population Health Sciences Institute Newcastle University Newcastle upon Tyne United Kingdom; 3 Faculty of Medicine and Health University of Sydney Sydney Australia; 4 Department of Computer and Information Sciences Northumbria University Newcastle upon Tyne United Kingdom; 5 Faculty of Medical Sciences The Medical School Newcastle University Newcastle upon Tyne United Kingdom

**Keywords:** accelerometry, digital technology, machine learning, physical activity, type 2 diabetes, digital biomarkers, digital phenotyping, mobile phone

## Abstract

**Background:**

Between 2013 and 2015, the UK Biobank collected accelerometer traces from 103,712 volunteers aged between 40 and 69 years using wrist-worn triaxial accelerometers for 1 week. This data set has been used in the past to verify that individuals with chronic diseases exhibit reduced activity levels compared with healthy populations. However, the data set is likely to be noisy, as the devices were allocated to participants without a set of inclusion criteria, and the traces reflect free-living conditions.

**Objective:**

This study aims to determine the extent to which accelerometer traces can be used to distinguish individuals with type 2 diabetes (T2D) from normoglycemic controls and to quantify their limitations.

**Methods:**

Machine learning classifiers were trained using different feature sets to segregate individuals with T2D from normoglycemic individuals. Multiple criteria, based on a combination of self-assessment UK Biobank variables and primary care health records linked to UK Biobank participants, were used to identify 3103 individuals with T2D in this population. The remaining nondiabetic 19,852 participants were further scored on their physical activity impairment severity based on other conditions found in their primary care data, and those deemed likely physically impaired at the time were excluded. Physical activity features were first extracted from the raw accelerometer traces data set for each participant using an algorithm that extends the previously developed Biobank Accelerometry Analysis toolkit from Oxford University. These features were complemented by a selected collection of sociodemographic and lifestyle features available from UK Biobank.

**Results:**

We tested 3 types of classifiers, with an area under the receiver operating characteristic curve (AUC) close to 0.86 (95% CI 0.85-0.87) for all 3 classifiers and F1 scores in the range of 0.80-0.82 for T2D-positive individuals and 0.73-0.74 for T2D-negative controls. Results obtained using nonphysically impaired controls were compared with highly physically impaired controls to test the hypothesis that nondiabetic conditions reduce classifier performance. Models built using a training set that included highly impaired controls with other conditions had worse performance (AUC 0.75-0.77; 95% CI 0.74-0.78; F1 scores in the range of 0.76-0.77 for T2D positives and 0.63-0.65 for controls).

**Conclusions:**

Granular measures of free-living physical activity can be used to successfully train machine learning models that are able to discriminate between individuals with T2D and normoglycemic controls, although with limitations because of the intrinsic noise in the data sets. From a broader clinical perspective, these findings motivate further research into the use of physical activity traces as a means of screening individuals at risk of diabetes and for early detection, in conjunction with routinely used risk scores, provided that appropriate quality control is enforced on the data collection protocol.

## Introduction

### The UK Biobank

Objective measures of physical activity can be used to characterize people’s free-living movement behavior to provide the kind of digital phenotype [1] that promises to support a vision of participatory, preventive, and personalized health care. The UK Biobank collected the largest available data set of free-living physical activity traces [2]. It includes uncontrolled, raw accelerometry traces collected for 7 days for a random selection of 103,712 out of a total of 502,664 UK Biobank participants (approximately 25%) between February 2013 and December 2015. All the studies cited here, including the one described in this paper, have used a reduced set after performing quality checks.

This data set has been used in recent studies to quantify differences in physical activity levels across the general UK Biobank population [3] and to show that participants with chronic diseases exhibit lower levels of activity than the general UK Biobank cohort [4]. It has also demonstrated associations between cardiometabolic health, multimorbidity, and mortality [5,6]. However, this data set has not been used to validate the hypothesis that accelerometer traces measures of physical activity can be used as a predictor for type 2 diabetes (T2D) and, thus, potentially, as a valid digital phenotype for early detection of T2D.

### T2D and Physical Activity

T2D is linked with low physical activity levels and increasing age [7]. This disease has become much more prevalent and is rapidly rising globally, especially in parts of the developing world [8].

Research into the effectiveness of activity monitoring for T2D detection and prevention is motivated by the disproportionately high cost, both economic and social, of treating T2D [9], considering that approximately 90%-95% of diagnosed diabetes among adults is type 2. In the United Kingdom alone, more than 2.7 million people have been diagnosed with T2D, whereas a further 750,000 people are believed to have the symptoms but are yet to be diagnosed with the disease [10].

Studies have been undertaken to use digital phenotypes for early diagnosis, but most studies have focused on using traditional multi-omics approaches [11].

### The UK Biobank Accelerometer Data and T2D

In this study, we tested the hypothesis that activity profiles, when represented in sufficient detail, differ significantly between individuals with T2D and the general population.

This study begins by defining participants with T2D in the UK Biobank using a combination of preexisting diagnoses collected in the UK Biobank assessment centers and automated analysis of the participants’ electronic health records (EHRs) follow-up. We then evaluate the extent to which accelerometer traces can distinguish individuals with T2D from normoglycemic controls. The approach employs a combination of traditional machine learning classification models to quantify the predictive power of features extracted from accelerometer traces and to assess their limitations relative to this task.

## Methods

### Overview

This paper refers to each volunteer’s 1-week activity recording period as their *wear time* and to the UK Biobank volunteers as the *accelerometry cohort*.

The data set used in this study was derived from the collection of activity traces for each of these participants, filtered using the inclusion and exclusion criteria described below. Variables representing physical activity features were extracted from the raw traces. In addition, a small set of sociodemographic, anthropometric, and metabolic variables were added, following recent studies [11] in which the same variables were used to characterize the behavioral phenotype of UK Biobank participants relative to cardiovascular disease (CVD) and T2D.

### Inclusion and Exclusion Criteria for T2D-Positive Participants

**Figure 1:**
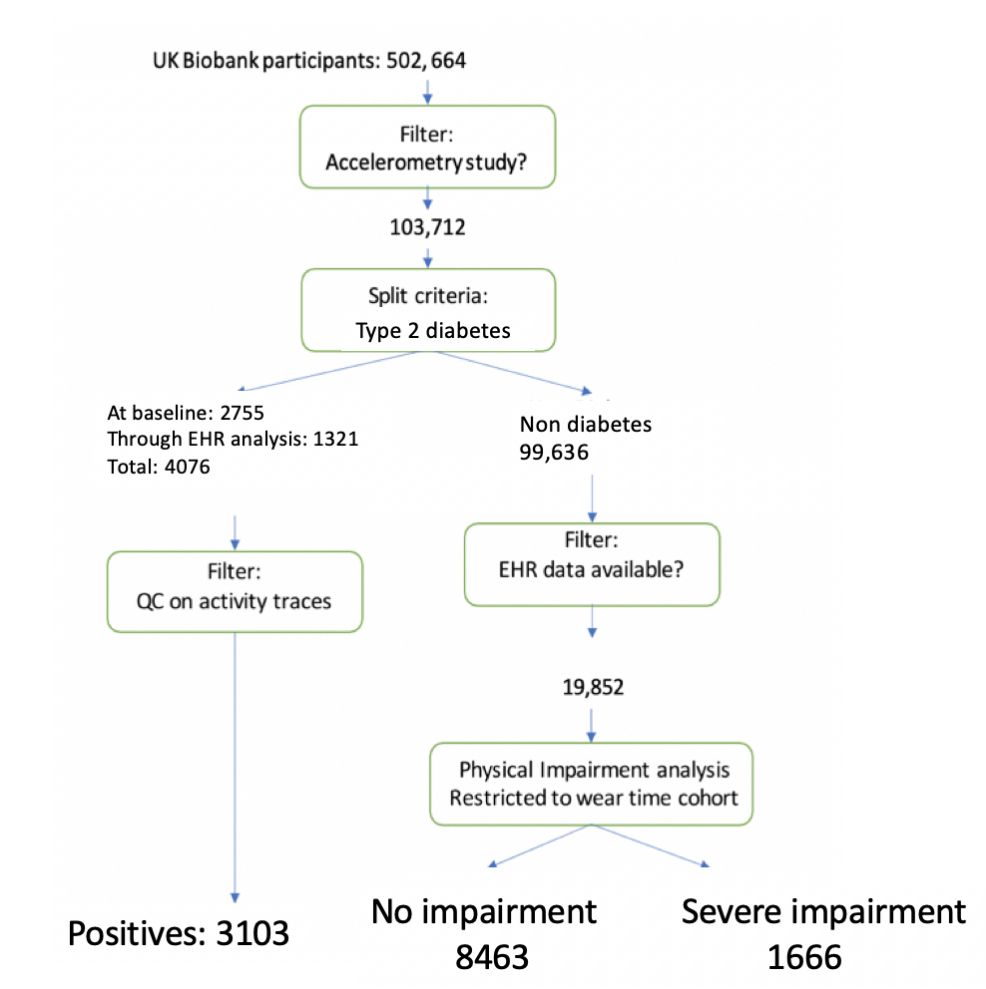
Training set selection criteria for type 2 diabetes–negative and type 2 diabetes–positive individuals. EHR: electronic health record; QC: quality control.

The criteria described below and the resulting data set sizes are summarized in Figure 1. Participants with T2D were identified using a combination of self-reported data collected at the Biobank assessment center and data from the participants’ primary care EHR, including prescriptions. At the time of writing, EHR records were available for approximately 245,000 out of 502,664 individuals (approximately 45%) of the UK Biobank population. Inclusion in the T2D group, based on self-reporting, follows the same criteria as in the study by Schüssler-Fiorenza Rose et al [11], namely, individuals with an explicit diagnosis as part of their assessment, based on the UK Biobank Showcase [12].

At the baseline assessment center, participants who had been diagnosed with diabetes or T2D were selected; those taking insulin within their first year (variable 2986-0.0) and who were less than 35 years old (variable 2976-0.0) at diagnosis were excluded to reduce the likelihood of individuals with type 1 diabetes and monogenic forms of diabetes [13]. This resulted in 2755 participants from the accelerometry cohort being identified as having T2D.

Primary care EHRs were also used to identify participants who developed T2D after their baseline assessment but before their accelerometer wear time. The incidence of T2D was defined as the occurrence of a Read Code version 2 or Clinical Terms Version 3 (CTV3) code corresponding to T2D after the date of the assessment center visit. Read Code version 2 code sets developed by Kuan et al were used [14], and equivalent CTV3 codes were mapped using mapping data provided by the UK Biobank [4,5].

The low prevalence of T2D in the UK Biobank population is reflected in the very small positive group, compared with an overwhelmingly large non-T2D control group (99,636 participants). Therefore, it is necessary to rebalance these classes before model learning. Rather than random selection from the control group, better selection criteria can be adopted.

We observed that the normoglycemic control group might include individuals with nondiabetes-related physical activity impairments. Excluding such individuals is desirable, as it is likely to remove noise from the control group. The controls’ selection process described below includes a judgment, grounded in general medical knowledge, of how a wide variety of conditions may have affected a participant’s ability to perform normal activities. Although the assessment may not be entirely accurate, to the best of our knowledge, this is the first attempt to select a control group based on EHR data. The outcome was assumed to be no worse than random selection from the control group. The results show that the prediction accuracy improves relative to using a random control training set.

The selection process involved a further analysis of EHRs for a period antecedent of wear time to identify any nondiabetes medical conditions that may have resulted in physical activity impairment. This analysis is limited by the partial availability of EHRs (approximately 20,000 individuals within the cohort). The analysis is described in detail in Multimedia Appendix 1. An impairment score is calculated for each individual by (1) associating a *severity score* with each type of relevant disease reference in the Read Code version 2 catalog and (2) averaging the scores across all occurrences of the disease references in the individual’s EHR history, within 6 months before wear time. Records are included for 1 month after wear time, as there may be a delay in recording new conditions. The analysis resulted in 2 control subpopulations, as shown in Figure 1 (bottom right): *Norm-0*, where we expected no impairment (n=8463), and *Norm-2*, with expected high impairment (n=1666). These results are summarized in Table 1. Both sets were used as part of supervised learning in separate experiments, as explained below.

**Table 1:** Number of participants in each subpopulation according to activity impairment severity score.

Impairment score	Total participants, N	Participants with adequate wear time, n (%)
Norm-0	11,019	8463 (76.80)
Norm-2	3355	1666 (49.66)

It is also acknowledged that 151 out of 3101 T2D-positive individuals also had a high impairment severity score for physical activity. This small subset of the T2D-positive population was not excluded from the training data sets. T2D is a complex disease that can cause many complications or comorbidity with other conditions, such as CVD. Therefore, to capture all behaviors and activity patterns associated with T2D, it is important to include the severely impaired T2D-positive individuals in the overall T2D-positive population.

We have also experimentally verified that removing these few individuals from the training set does not alter the properties of the resulting model (refer to the *Results* section).

### Training Data Sets

Using these 2 control groups, 2 training sets were formed: training set 1: T2D versus Norm-0 and training set 2: T2D versus Norm-2. The first was used to test our main hypothesis that activity levels in the T2D group were significantly different from those in the unimpaired control group. The second was used to quantify the effect of possible nondiabetic activity impairment as a source of noise in the controls. This was achieved by training the same models using training set 1 and training set 2 and then comparing their relative predictive performance.

### Physical Activity Features

**Figure 2:**
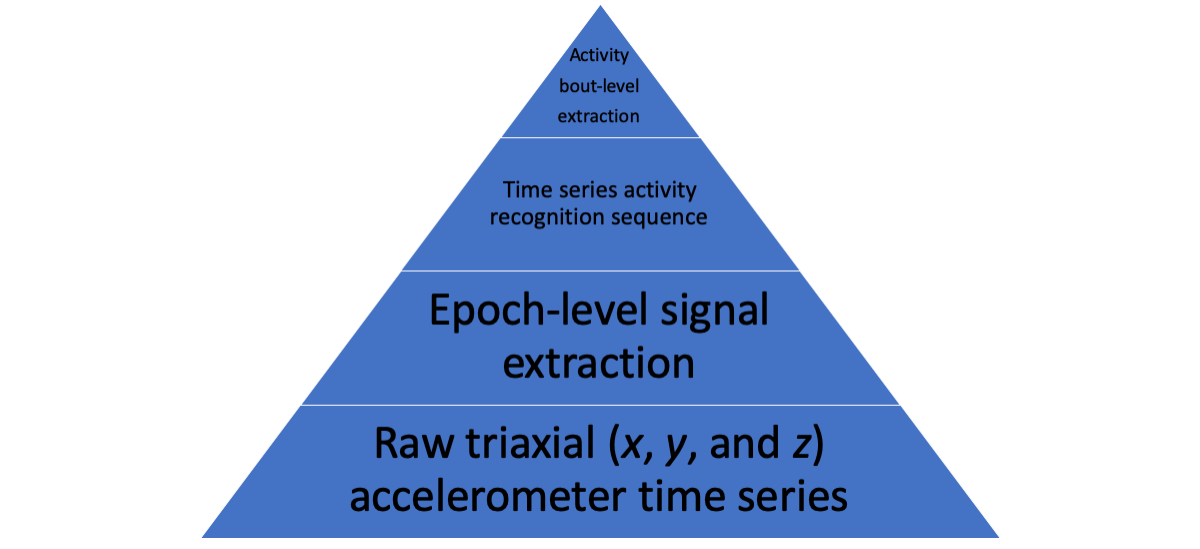
Hierarchy of physical activity representations.

A raw accelerometry trace consists of a triaxial (*x*, *y*, and *z*) time series. The open-source accelerometer analysis toolkit developed at the University of Oxford, available on GitHub [15], was used to annotate timelines for each raw activity trace [16]. The tool breaks down the time series into 30-second fragments, called *epochs*, and then employs a classifier (random forests and hidden Markov models) to annotate a time series in which each epoch belongs to 1 of 5 activity types: *sedentary*, *moderate*, *walking*, *sleep*, and *light tasks*. This tool distinguishes between walking from sedentary and moderate activities. According to the authors of this study, these activity types correspond to the following metabolic equivalent of task levels: sedentary, 1.5; moderate, 4.9; walking, 3.2; sleep, 1.0; and light tasks, 2.2. The feature extraction hierarchy is summarized in Figure 2.

The level above the time series activity recognition sequence uses activity bouts. An activity bout is defined as a single epoch or an uninterrupted consecutive series of epochs in which a single activity type is performed. The length of a bout refers to the many 30-second epochs for which each bout is performed. The features extracted for this study are at the level of activity bouts of each activity type: their frequency, average length, and percentage of time spent in each, broken down into fractions of a 24-hour day. This choice is inspired by neuroscience research on the effects of cognitive impairment in early stages of Parkinson disease on gait, where ambulatory bouts play a key role [17,18]. A personalized analysis of daily activities was performed to extract these features. First, to accommodate for different sleeping habits, night-sleep time boundaries were identified for each individual. These are defined as the average of the largest nearly continuous period of sleep activity bouts over a 24-hour period. The remaining period of the 24-hour day is then divided into 3 phases, denoted as morning, afternoon, and evening. Within each phase, the activity bout level features were extracted for each activity type.

**Figure 3:**
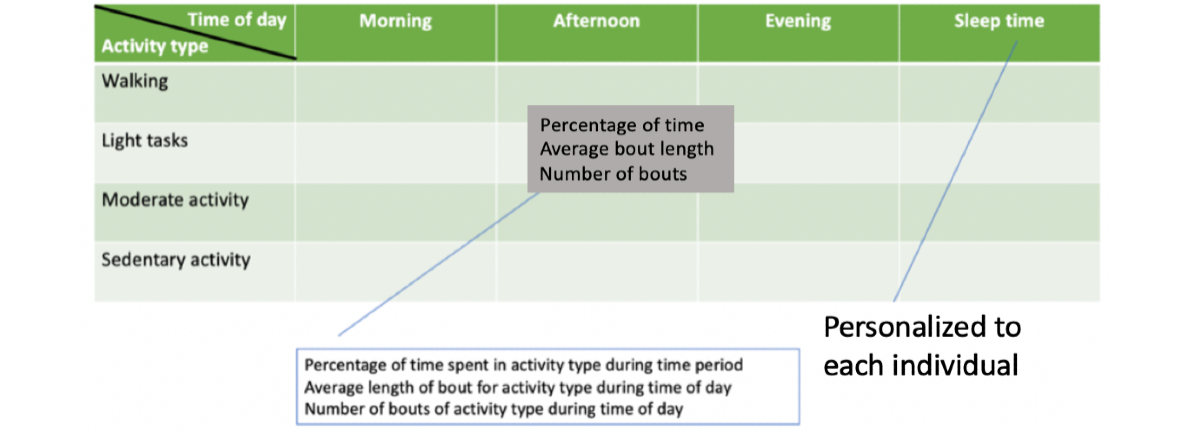
Feature matrix for physical activity bout representation space.

This analysis results in a breakdown of 60 activity bout-level features, organized into a 5×4 matrix for each individual, with features extracted for four periods of the 24-hour day including sleep time as shown in Figure 3. Each element in the matrix (the type of activity and time of day) has 3 features: (1) number of bouts for that activity, (2) percentage of time spent in the activity, and (3) average length of the bouts. This arrangement resulted in a total of 60 features per individual. These were then aggregated over 7 days of wear time, taking the average for each element in the matrix. This feature space is referred to as the *high-level activity bout features* in this study. The code is available on GitHub [19].

### Sociodemographic, Anthropometric, and Lifestyle Features

To quantify the relative importance of the new high-level activity bout features when used in machine learning, traditional sociodemographic and lifestyle indicators that are commonly associated with the incidence of T2D have been added. These are shown in Table 2 and were chosen based on previous studies [5,20]. These features are combined with self-reported physical activity assessments, some of which are not part of the output from the Oxford accelerometer analysis tool, notably vigorous activity. In contrast, the physical activity features in our approach are the high-level activity bout features obtained from objective accelerometer measurements. Objective physical activity metrics also help to validate subjective measurements [21,22].

**Table 2:** Sociodemographic, lifestyle, and anthropometric characteristics selected from the UK Biobank baseline assessment for comparison with high-level activity bout features space.

Sociodemographic, lifestyle, and anthropometry characteristic	Description
Sex	Male or female (approximately 50:50 ratio)
Age at the assessment center	Recruits at baseline were aged between 40 and 69 years
Ethnic group	Predominantly White British, with some participants identifying as Black, Asian and Minority Ethnic groups
Alcohol drinking status	Participant reports if they were alcohol drinkers in the past, were currently drinking alcohol, or never had drunk alcohol
Smoking status	Participants report if they had smoked in the past, were currently smoking, or had never smoked
Body fat percentage	Percentage of fat in total body mass (a better indicator for obesity than BMI)
Waist circumference	Measurement taken around the abdomen at the level of the umbilicus (belly button)
Sleep duration	Self-reported average duration of sleep in a day
Time spent watching television	Self-reported average time spent watching television per day
Townsend index	Metric for material deprivation within a population
Duration of walking activity	Self-reported average duration of time spent walking in a day
Duration of vigorous activity	Self-reported average duration of time spent performing vigorous activities during the day
Duration of moderate activity	Self-reported average duration of time spent performing moderate activity during the day

The International Physical Activity Questionnaire-Short Form was used for the variables measuring physical activity (including moderate, vigorous, and walking), television viewing times, and sleep duration (Table 2). Some of these sociodemographic and lifestyle features contained missing data. This was solved using a k-nearest neighbor imputer in scikit-learn [23], which calculates the missing value using the mean of k-nearest neighbors found in the training data using Euclidean distances, thus preserving the distribution of the original data.

### Binary Classification

This exercise compares a number of classification models, obtained using different learning algorithms and using training sets training set 1 and training set 2, introduced earlier, in separate sets of experiments. Furthermore, different combinations of features were considered for each of the training sets: (1) high-level activity bout features only, (2) sociodemographic and lifestyle features only, and (3) high-level activity bout features combined with sociodemographic and lifestyle features.

These combinations produce a space of 6 data sets on which the models are trained. Three learning algorithms were tested on these data sets: random forest, logistic regression, and Extreme Gradient Boosting (XGBoost) algorithm. XGBoost is a relatively recent and perhaps less known algorithm [24], which has come to prominence owing to its superior performance, both in terms of training time and prediction accuracy, compared with random forests. XGBoost uses gradient boosting, an ensemble method that builds a stronger classifier by adding weaker models on top of each, iteratively, until the training data achieve a good level of prediction performance.

A total of 18 classifier models were trained using these combinations of 6 data sets and 3 algorithms. A standard 10-fold cross-validation was used to avoid overfitting. When learning the classifiers, a random selection of half the Norm-0 T2D-negative controls in training set 1 only was undertaken to balance the size of the Norm-0 T2D-negatives and T2D-positive (3103 individuals). Norm-0 T2D-negative individuals still vastly outnumbered the T2D-positive population.

Following common practice for binary classifiers, this study reports F1 scores, precision, recall, and area under the receiver operating characteristic curve (AUC) scores. F1 conveys the balance between precision and recall and is a value between 0 and 1, where 1 indicates perfect precision and recall. It is calculated using the harmonic mean of the precision and recall. The AUC is a metric, with values between 0 and 1, for how well a classifier is capable of distinguishing between 2 classes. A value of 1 implies a good measure of discrimination, whereas a value of 0.5 implies no discrimination capacity.

On the basis of these performance and evaluation metrics, models were compared to assess (1) the differences in predictive power between the 2 feature sets using training set 1; (2) the effect of noise in controls, using training set 2; and (3) the best modeling algorithms.

### Clustering Analysis

Further analysis was undertaken where unsupervised clustering algorithms were used to segregate and identify unlabeled individuals that exhibit similar behavior with the new high-level activity bout feature space. These clusters were then profiled and interpreted in terms of their anthropometric, lifestyle, and sociodemographic characteristics. This analysis is beyond the scope of this paper but is reported in Multimedia Appendix 2.

## Results

### Distribution of Physical Activity Features

To summarize the distribution of the T2D-positive and Norm-0 T2D-negative populations, the high-level activity bout features were aggregated for a 24-hour period and averaged across both populations.

On average, both the T2D-positive and T2D-negative populations do not undertake significantly different quantities of each activity type aggregated to the level of the 24-hour day with approximately 5% moderate activity, 42% sedentary activity, 38% asleep, 5% light tasks, and 10% walking. However, the high-level activity bout features also offer an insight into the regularity and length of activity bouts. The values for these features do offer some discrimination between the T2D-positive and Norm-0 T2D-negative populations. The histograms below demonstrate an example of this by showing the distribution of daily averages for bout length, the number of bouts, and the percentage of times spent on sleep activity.

**Figure 4:**
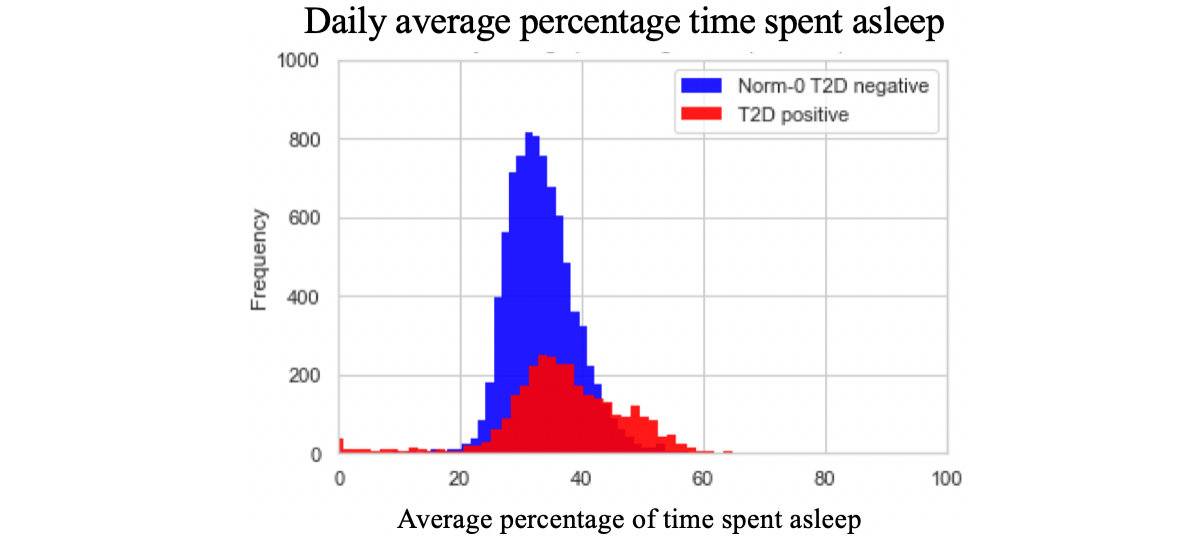
Histogram for daily average percentage times spent asleep.

**Figure 5:**
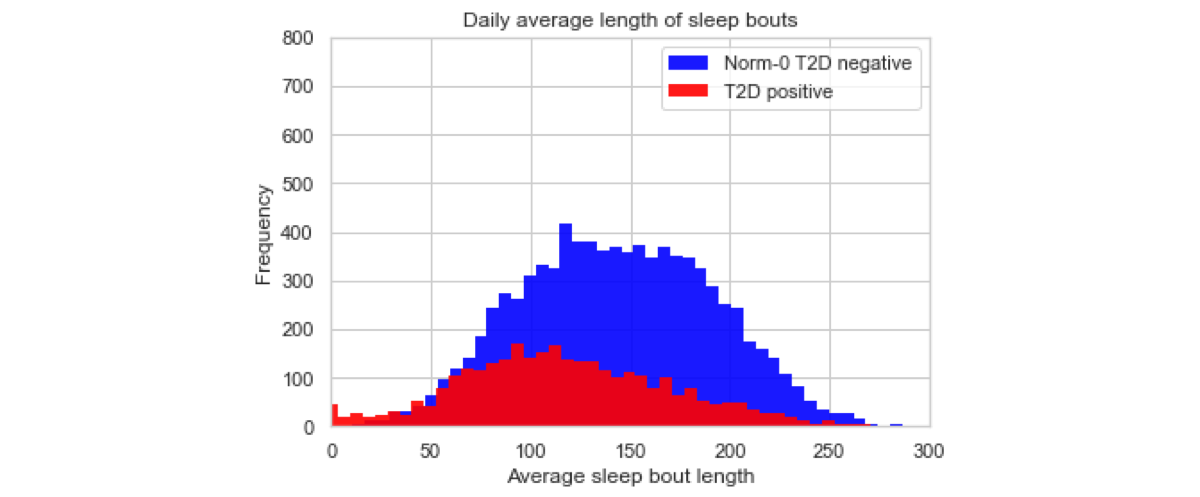
Histogram for daily average length of sleep bouts.

**Figure 6:**
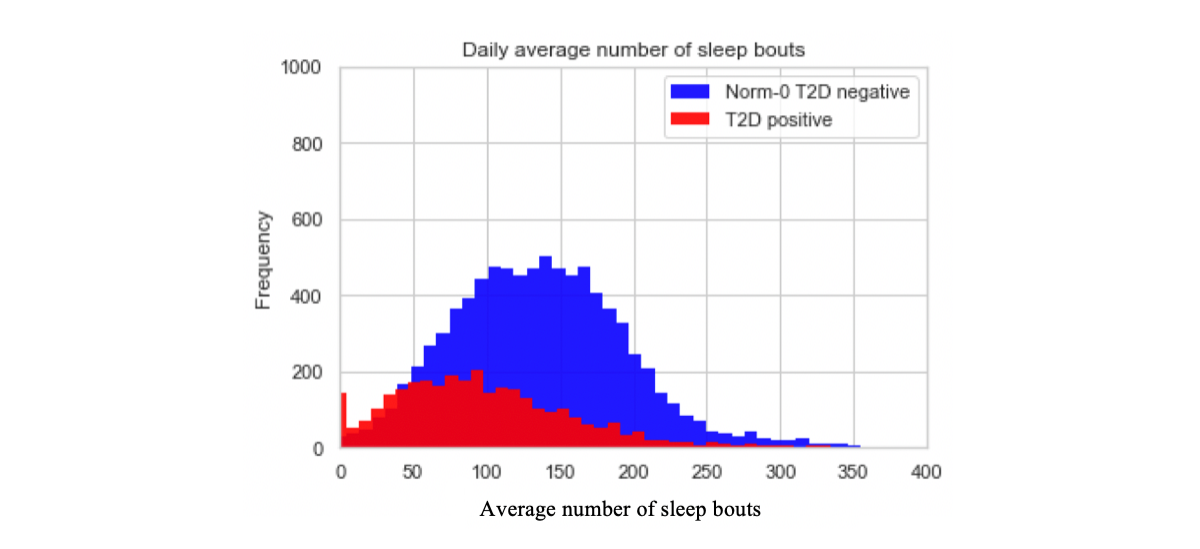
Histogram for daily average number of sleep bouts.

The histograms in Figures 4-6 show noticeable differences between the 2 populations in the features that we have developed, when aggregated out to a day. Breaking the daily patterns into 4 distinct times of day (morning, afternoon, evening, and during sleep) would further demonstrate the differences in activity bout patterns for the 2 populations by virtue of the granularity. The combined effect of all these granular-level activity bout features produces high model accuracy, as reported below.

### Binary Classification

**Figure 7:**
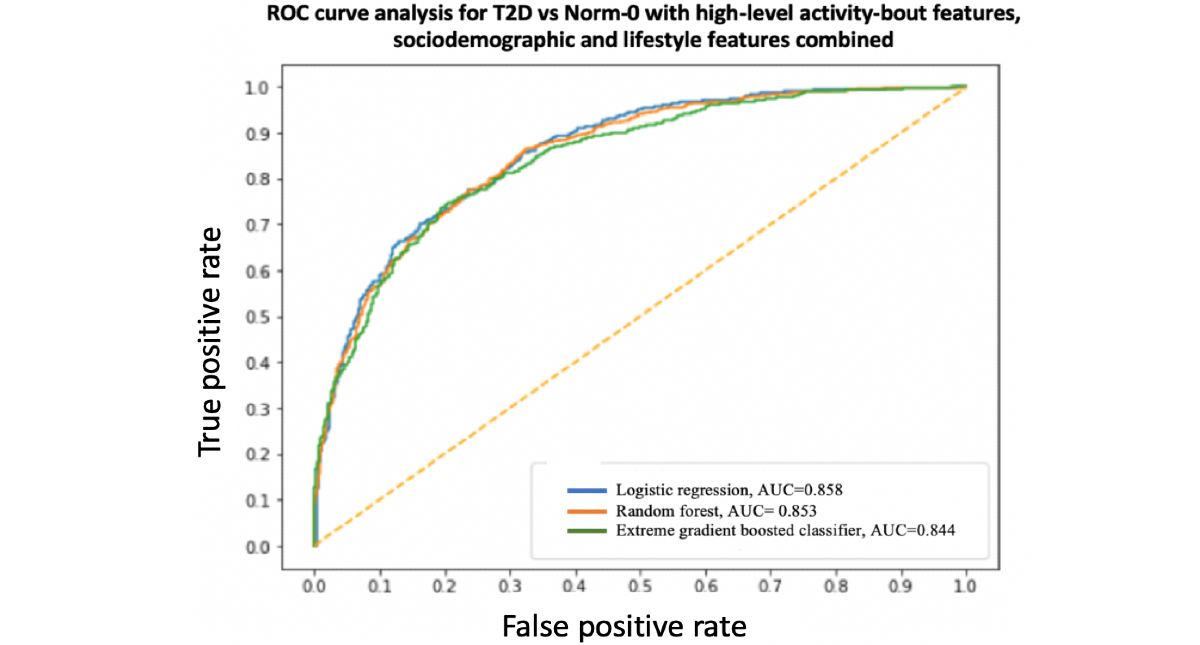
Receiver operating characteristic curve and area under the receiver operating characteristic curve for type 2 diabetes vs Norm-0: High-level activity bout features & sociodemographic and lifestyle features combined. AUC: area under the receiver operating characteristic curve; ROC: receiver operating characteristic curve; T2D: type 2 diabetes.

**Figure 8:**
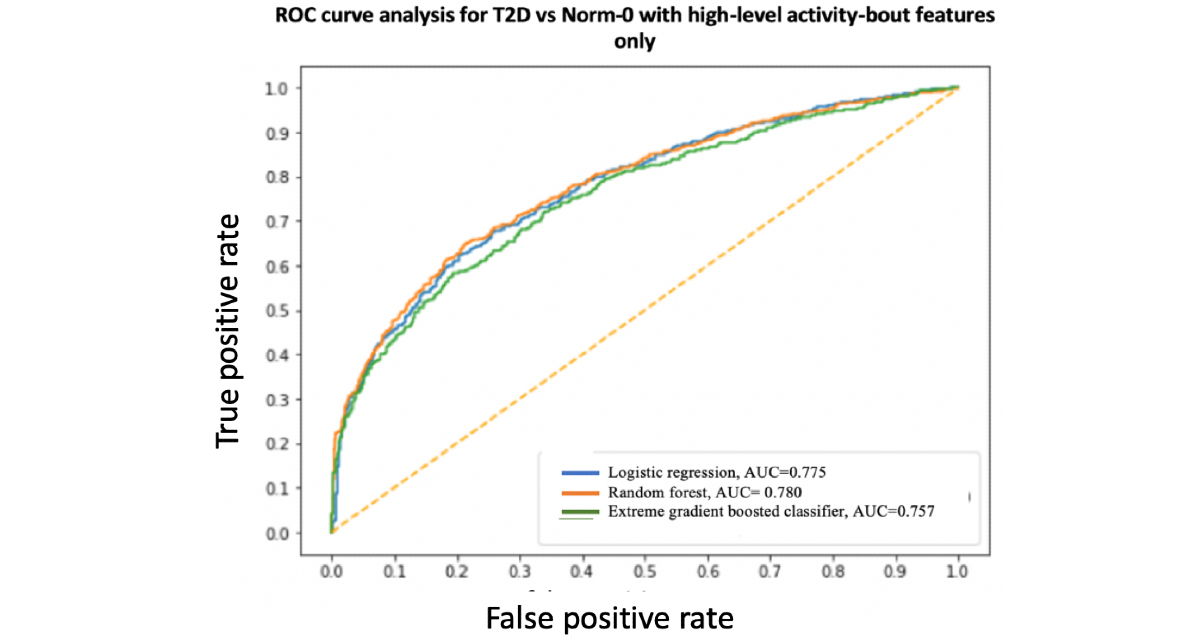
Receiver operating characteristic curve and area under the receiver operating characteristic curve for type 2 diabetes vs Norm-0: High-level activity bout features only. AUC: area under the receiver operating characteristic curve; ROC: receiver operating characteristic curve; T2D: type 2 diabetes.

**Figure 9:**
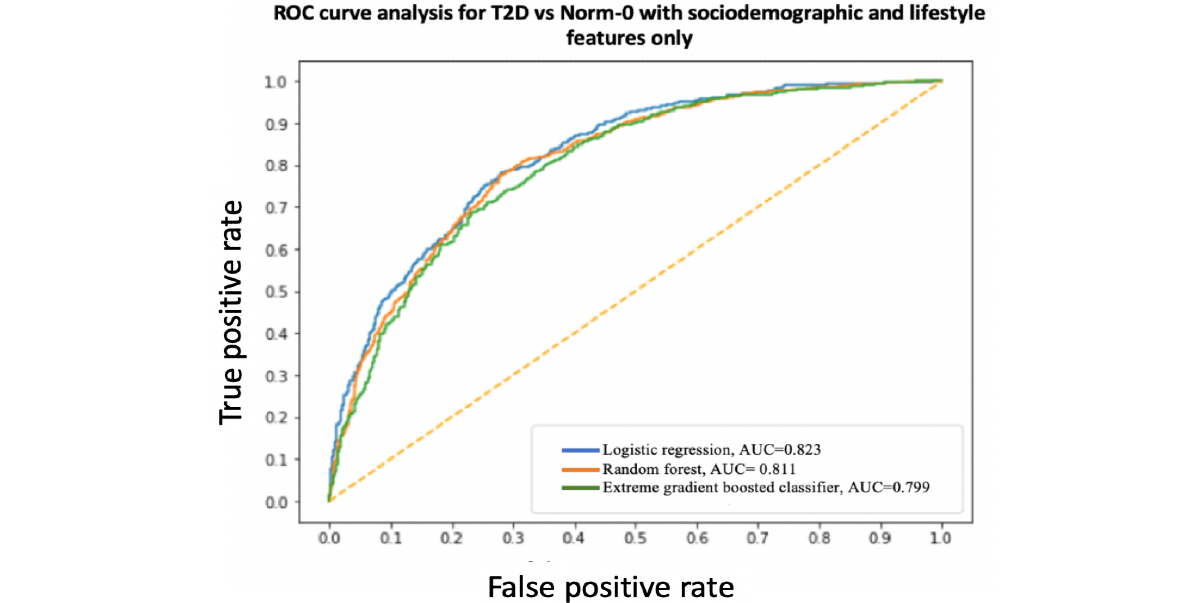
Receiver operating characteristic curve and area under the receiver operating characteristic curve for type 2 diabetes vs Norm-0: Sociodemographic and lifestyle features only. AUC: area under the receiver operating characteristic curve; ROC: receiver operating characteristic curve; T2D: type 2 diabetes.

**Figure 10:**
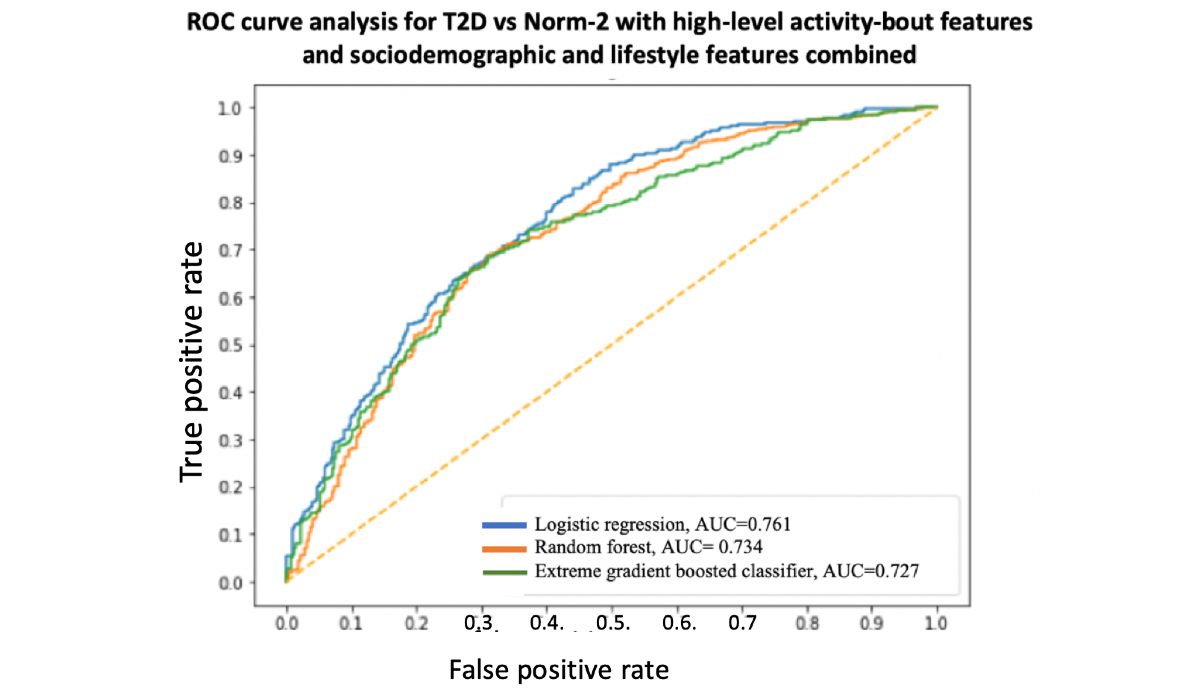
Receiver operating characteristic curve and area under the receiver operating characteristic curve for type 2 diabetes vs Norm-2: High-level activity bout features & sociodemographic and lifestyle features combined. AUC: area under the receiver operating characteristic curve; ROC: receiver operating characteristic curve; T2D: type 2 diabetes.

**Figure 11:**
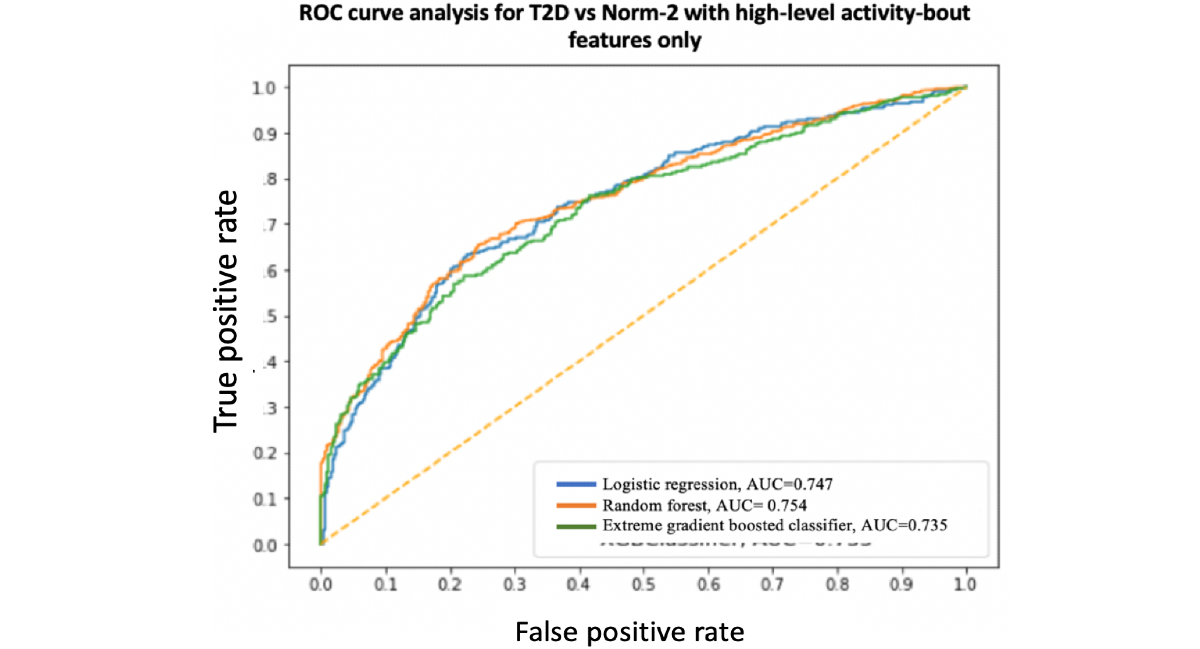
Receiver operating characteristic curve and area under the receiver operating characteristic curve for type 2 diabetes vs Norm-2: High-level activity bout features only. AUC: area under the receiver operating characteristic curve; ROC: receiver operating characteristic curve; T2D: type 2 diabetes.

**Figure 12:**
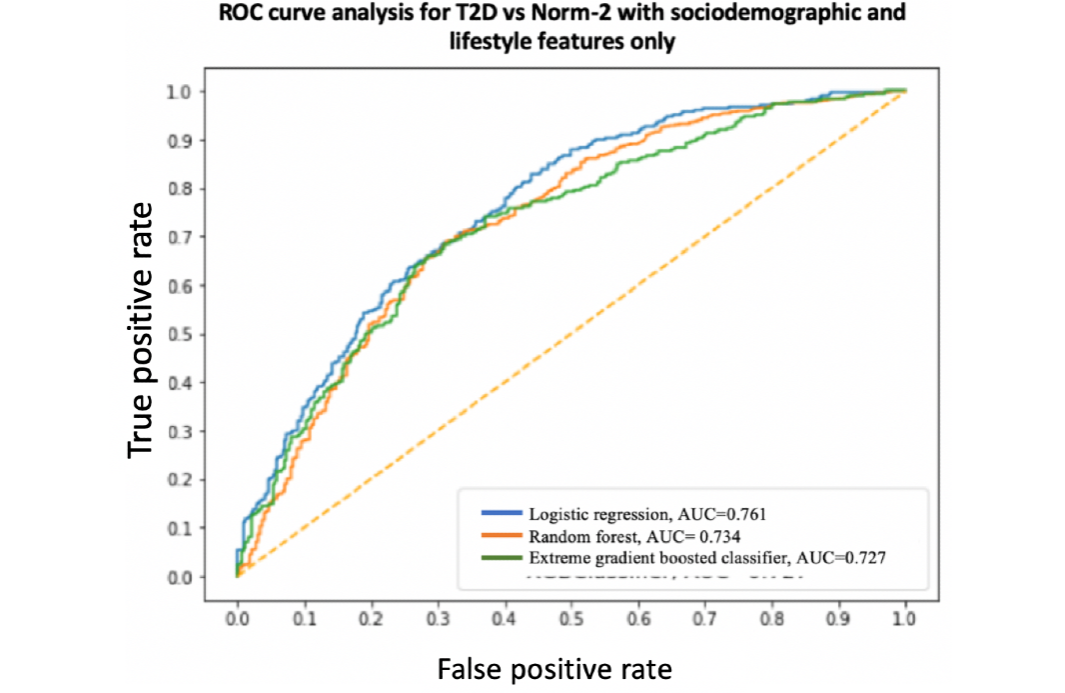
Receiver operating characteristic curve and area under the receiver operating characteristic curve for type 2 diabetes vs Norm-2: Sociodemographic and lifestyle features only. AUC: area under the receiver operating characteristic curve; ROC: receiver operating characteristic curve; T2D: type 2 diabetes.

A summary and performance comparison across the 18 models built for this study is presented in Tables 3 and 4, where AUC measures are obtained by averaging over 10 models using cross-validation for robustness. The receiver operating characteristic (ROC) curves and AUC scores are shown in Figures 7-12. All models were split between training and test data sets with an 80:20 ratio. More detailed metrics for precision, recall, F1, and ROC curves, using 10-fold cross-validation, are available in Multimedia Appendix 3.

**Table 3:** Classification results measured using area under the receiver operating characteristic curve scores, showing the effect of choice of type 2 diabetes–negatives, Norm-0 (no physical activity impairment) versus Norm-2 (severe physical activity impairment). The values in the cells represent area under the receiver operating characteristic curve scores.

Predictive model	High-level activity-bout features		Sociodemographic and lifestyle		High-level activity bout features+sociodemographic and lifestyle	
	Norm-0	Norm-2	Norm-0	Norm-2	Norm-0	Norm-2
Random forest	0.80	0.68	0.83	0.78	0.86	0.77
Logistic regression	0.79	0.70	0.83	0.78	0.86	0.78
Extreme gradient boosting	0.78	0.66	0.80	0.74	0.85	0.75

**Table 4:** Classification results measured using F1, showing the effect of choice of type 2 diabetes-negatives, Norm-0 (no physical activity impairment) versus Norm-2 (severe physical activity impairment). The values in the cells represent F1 scores.

Predictive model and T2D^a^ status		High-level activity bout features		Sociodemographic and lifestyle		High-level activity bout features+sociodemographic and lifestyle	
		Norm-0	Norm-2	Norm-0	Norm-2	Norm-0	Norm-2
**Random forest**							
	T2D-positive	0.65	0.70	0.65	0.77	0.73	0.77
	T2D-negative	0.78	0.54	0.78	0.63	0.81	0.63
**Logistic regression**							
	T2D-positive	0.66	0.72	0.69	0.77	0.74	0.77
	T2D-negative	0.77	0.54	0.79	0.65	0.82	0.65
**Extreme gradient boosting**							
	T2D-positive	0.66	0.68	0.67	0.74	0.73	0.76
	T2D- negative	0.77	0.52	0.76	0.62	0.80	0.63

^a^T2D: type 2 diabetes.

When performance is measured using AUC, stronger results are achieved when using high-level activity bout features and sociodemographic and lifestyle in combination, as expected. Using high-level activity bout features on their own reduces performance (approximately 7%-8%). However, high-level activity bout features provide almost the same performance as traditional sociodemographic and lifestyle features on their own.

Models were also generated using alternate training data sets, where 151 T2D-positive individuals with high physical activity impairment severity scores were excluded. These models exhibit very similar performance to those presented above, suggesting that physically impaired (Norm-2) T2D-positive individuals can be used as part of the T2D positives in the training set.

F1 measures in Multimedia Appendix 3 reveal differences in classification accuracy between T2D against Norm-0 controls, and T2D against Norm-2 controls. When using Norm-0 controls, negatives are more accurately predicted than T2D, presumably because of class imbalance (4178 vs 3103). It is also clear that excluding physically impaired negatives improves the results.

When Norm-2 is used, however, T2D is more accurately predicted than negatives, perhaps because in this case, Norm-2 is the minority class (1666 vs 3103) and because of potential diversity within the highly impaired control population. This will be investigated in a future study.

In all cases, the combination of high-level activity bout features and sociodemographic and lifestyle variables gives better results than using either set of features on their own, as expected. The performances of both feature sets are largely independent of the choice of the learning algorithm, as seen by the overlapping ROC curves.

## Discussion

### Principal Findings

Using data from the UK Biobank, this study supports the hypothesis that individuals with diagnosed T2D exhibit physical activity patterns that are significantly different from those of normoglycemic controls, thus providing novel ways to detect T2D, that is, through appropriate analysis of physical activity patterns. Although most previous studies, particularly using UK Biobank, are limited to self-reported physical activity levels [5,11,25], here we have demonstrated the benefits of extracting a more objective and granular representation of physical activity from raw accelerometry traces data, namely, by activity type and time of day or sleep time. Using these features, either on their own or in combination with a selected set of sociodemographic, anthropometric, and lifestyle variables, we have shown that appropriately trained machine learning models were able to discriminate between the 2 cohorts with good predictive accuracy.

### Practical Significance

These findings suggest that it may be possible to use continuous or periodic self-monitoring of individuals at risk of T2D, specifically those in a prediabetes state, for screening and early detection of disease progression. This is particularly important as evidence shows that reversal of T2D is possible, with a higher success rate when interventions are undertaken within the first 5 years of the disease [26-28].

However, early detection is still an unsolved problem, with recent figures reporting that over 190 million people worldwide live with undiagnosed diabetes [29]. Risk scores that are routinely used for screening, such as the Leicester score, are easy to obtain but not very accurate [30].

This suggests that self-monitoring of physical activity patterns, such as those presented in this study, may complement risk scores to help with the early detection of T2D, especially in high-risk individuals. Today, this can be achieved at a low cost using readily available technology [31], including internet-enabled data loggers that do not require participants to return devices, such as smartphones, periodically. However, further research is required to establish the quality and significance of physical activity data for this specific purpose.

### Limitations

In principle, it may be possible to try and detect early signs of T2D using specific *fingerprint* patterns found in physical activity traces, where an example of a pattern may be *a person who takes short bouts of low or moderate activities with frequent sedentary breaks in between*. However, in practice, we found no evidence in the UK Biobank data set that strong correlations exist between specific physical activity patterns and T2D. Thus, what the machine learning approach has to offer may be limited to the strong indication demonstrated in this work, namely, that granular features extracted from the raw traces, taken together, are indeed good predictors and usefully augment the more traditional sociodemographic set of variables.

Although the UK Biobank is the largest known public accelerometry data set where a T2D cohort can be identified, detecting differences between T2D and controls remains challenging because of their low prevalence in the population, which is reflected in this study with the relatively small data set available for training when using supervised machine learning. Simultaneously, this data set was subject to noise for two reasons. First, because no formal quality assurance protocol was enforced during data collection, and second, because of the limited knowledge about other non-T2D–related conditions among the controls, which may contribute to reduced physical mobility or a more sedentary routine. We have shown how EHRs can be used to overcome this limitation.

### Conclusions

This study motivates further research into the use of granular physical activity measures as a form of *digital phenotype* for T2D. It also suggests that more rigorous protocols on wearing physical activity loggers are required to improve the quality of the data and the signal-to-noise ratio, along with stringent inclusion and exclusion criteria or at least comprehensive knowledge of clinical conditions that may affect the signal in the traces. This is also reflected in other studies [32,33]. When such quality criteria are met, it should be possible to repeat the analysis presented here using data sets from large-scale deployment of physical activity loggers to validate the hypothesis that early detection of T2D is scientifically and technically feasible.

## Appendix

Multimedia Appendix 1 Activity impairment scoring details.

Multimedia Appendix 2 Analysis details and results of unsupervised clustering work.

Multimedia Appendix 3 Full results details of analysis.

## Abbreviations

AUC: area under the receiver operating characteristic curve
CTV3: Clinical Terms Version 3
CVD: cardiovascular disease
EHR: electronic health record
ROC: receiver operating characteristic curve
T2D: type 2 diabetes
XGBoost: Extreme Gradient Boosting
