# Supplementary material for: Using Wearable Activity Trackers to Predict Type 2 Diabetes: Machine Learning–Based Cross-sectional Study of the UK Biobank Accelerometer Cohort
Source: JMIR Diabetes. 2021 Mar 19;6(1):e23364. doi: 10.2196/23364 (PMC8080299; doi:10.2196/23364)
Supplement: Multimedia Appendix 1 [file diabetes_v6i1e23364_app1.pdf]

Multimedia Appendix A: Scoring UK Biobank accelerometer cohort physical activity impairment level using EHR phenotypes

Non-diabetes UKBB participants may suffer from other conditions that may impact their normal physical activity. If these conditions are known, the participants can be excluded from the negative portion of any training set to prevent noise. We have used the primary care Electronic Health Records available from the UK Biobank to identify medical conditions that occurred within an interval of time prior to the accelerometer wear time, and including a short period afterwards. We have then developed an *activity impact score* to express the potential impairment due to the condition. Controls are then selectively excluded from the control portion (negative class) of the training set by setting a threshold on the activity impact score.

To calculate the *activity impact score* for a person, we assigned individual scores to the Read v2 and Read v3 codes used to encode conditions as expressed in Electronic Health Records (EHR). For each participant, we then aggregated all scores corresponding to the conditions found in that participant's EHR history within a given time interval around the accelerometer wear time (1 year prior and 6 months after was used for these calculations).

Read v2 uses over 28,000 codes to catalogue clinical events, however these are organised hierarchically, starting from macro categories, called *chapters*. These are characterised by a "byte-depth", for example:

1-byte depth: 'C....' Endocrine, nutritional, metabolic and immunity disorders

2-byte depth: 'C2…' Nutritional deficiencies

3-byte depth: 'C24..' Vitamin A deficiency

4-byte depth: 'C247.' Vitamin A deficiency with other ocular manifestation

5-byte depth: 'C2470' Vitamin A deficiency with xerophthalmia

This organisation makes it possible to assign scores to whole categories with the desired precision. Codes were assigned mostly at byte-depth 2 or 3, and administrative (no- clinical) chapters were removed. This pruning resulted in the assignment of activity impact codes to 215 chapters. Online knowledge resources published by the NHS were used to assess the level of physical impairment for each of the chapters. These were encoded as 1: low/no impact, 10: medium impact or 100: high impact. An individual's total impact score is calculated as the average of all scores for the conditions found in their EHRs (default is 0).

It should be emphasised that the aim of this assignment of codes to individuals is to ensure minimal noise in the control group used to train our classifiers. Considering that the size of the controls is about 30 times that of the T2D (participants), the choice of controls can be very selective. To be conservative, we have chosen a non-linear curve that assigns a high impact score to individuals with few severe impairments and requires uniformly no impairment to achieve a low score.

Table 1 shows the complete list of chapters with the corresponding frequency of occurrence for a random sample of about 20,000 participants from the accelerometer cohort. This provides an indication of which chapter are common/rare.

Chapter	Individuals with code	% Individuals with code	Occurrences	Occurrences/individual

**Z**	6,304	31.75499	20,665	3.278077
**Y**	12,811	64.53254	168,085	13.12037
**X**	1	0.005037	1	1
**U**	571	2.876285	714	1.250438
**T**	3,713	18.70341	5,256	1.415567
**S**	10,444	52.60931	23,517	2.251723
**R**	8,883	44.74612	23,692	2.667117
**Q**	96	0.483578	102	1.0625
**P**	866	4.362281	1,099	1.269053
**N**	14,624	73.66512	71,189	4.867957
**M**	12,760	64.27564	44,515	3.488636
**L**	2,854	14.37639	5,382	1.885774
**K**	10,097	50.86137	31,726	3.142121
**J**	9,094	45.80899	25,108	2.760941
**H**	11,538	58.12009	43,961	3.810106
**G**	11,346	57.15293	50,680	4.466772
**F**	12,449	62.70905	43,134	3.464857
**E**	5,635	28.38505	15,959	2.832121
**D**	1,476	7.435019	2,526	1.711382
**C**	6,609	33.29136	22,535	3.409744
**B**	7,207	36.30365	14,914	2.069377
**A**	9,209	46.38827	20,747	2.252905
**_**	13,192	66.45174	134,033	10.16017
**.**	169	0.8513	257	1.52071
**9**	19,694	99.20411	830,373	42.16376
**8**	19,316	97.30002	553,941	28.67783
**7**	17,983	90.58533	140,799	7.829561
**6**	19,340	97.42091	647,865	33.49871
**5**	15,042	75.7707	77,150	5.128972
**4**	19,300	97.21942	4,945,710	256.2544
**3**	16,674	83.99154	136,647	8.195214

**2**	19,538	98.4183	1,388,026	71.04238
**1**	19,686	99.16381	636,217	32.31825
**0**	4,874	24.55168	7,105	1.457735

Table 1Read v2 chapters and number of individuals where each chapter code appears in their EHR

Read codes physical impairment impact analysis.

The assignment of impact scores to individual Read v2 chapters at a given byte-depth consists of several steps.

Firstly, it should be noted that Biobank uses a combination of Read v2 and Red v3 scores. Since Read v3 are not organised hierarchically, as a preliminary step we have mapped all Read v3 to Read v2, using the official cross-maps available at https://nhs-digital.citizenspace.com/uktc/crossmaps/.

The second step involves selecting which chapters are to be or not be assigned an activity score. The decisions are listed below, along with the rationale for the less obvious of those.

Excluded Chapters

0 – Occupation: not relevant, not easy to link codes to physical impairment.

1 – History/Symptoms. Broad chapter covering a vast number of different topics surrounding medical history. This is a very commonly used chapter (99.16% of individuals at an average occurrence of 31.32) and thus unlikely to be useful for selection.

5 – Radiology/Medical Physics. Holds codes surrounding X-rays, ultrasounds and other medical screenings. Potential elements of risk of impairment have to be found at level 4 or 5 and require specialised clinical knowledge.

8 – Other therapeutic procedures. This Is a broad chapter that covers Post Operation Monitoring to Physiotherapy, however it is very common (97.30% of individuals at an average occurrence of 28.68) and thus not useful for selection. Also not immediately relevant, as 30% of the entries in this whole chapter are concerned with Medication Review (8B3V.).

9 – Administration

L – Complications of pregnancy, childbirth and the puerperium. Excluded as the UKBB population starts at age 40 and this code applies to 14.37% of individuals at an average occurrence of 1.89.

P – Congenital anomalies. These are conditions present from birth, such as Heart Defects or Cleft Lips (4.36% of individuals at an average occurrence of 1.27). Given the age of the individuals of this study, this code was excluded as individuals with congenital anomalies are assumed to have adjusted their lifestyles accordingly at this approximately middle stage of life expectancy (40+ years).

Q – Perinatal conditions. Describes conditions immediately before or after giving birth (0.48% of individuals at an average occurrence of 1.06). Again excluded due to the age of the individuals in this study.

R – [D]Symptoms, signs and ill-defined conditions. Information too vague to be used confidently. Vague symptoms used mainly when the medical practitioner is unsure of a condition and wants to submit symptoms into the system.

T – Causes of injury. This is associated with chapter S (which is included), and this chapter simply holds the reason behind an entry.

U – [X]External causes of morbidity and mortality.

Z – Unspecified conditions. Not specific enough to be used.

Included Chapters

3 – Diagnostic Procedures
This is a broad chapter that covers a vast range of medical procedures or tests, Table 1 shows this chapter exists in 83.99% of individuals at an average occurrence of 8.2. However, it is difficult to understand why the procedure has been performed, One subchapter describes tests for disabilities, but the outcomes of these tests are not the main focus, because someone who is being tested is likely to have been diagnosed to have physical ailment using other codes, namely : Disability assessment – Physical (39***)

6 – Preventative Procedures

These can be vaccines, counselling and stroke prevention. Table 1 shows this exists in 97.42% of individuals with an average occurrence of 33.5. The following specific subchapters were included:

- Chronic disease – general (661**)
- Cardiac disease monitoring (662**)
- Respiratory disease monitoring (663**)
- Gout monitoring (669**)
- Diabetic monitoring (66A**)
- Obesity monitoring (66C**)
- Alcohol disorder monitoring (66e**)
- Cardiovascular disease monitoring (66f**)
- Arthritis monitoring (66H**)
- Chronic pain review (66n**)
- Further diabetic monitoring (66o**)
- Warfarin monitoring (66Q**)
- Lipid disorder monitoring (66X**)
- Primary Prevention ischaemic heart disease (6C***)
- Stroke prevention (6F***)

7 – Operations & Procedures

Table 1 shows this chapter exists in 90.58% of individuals at an average occurrence of 7.82. Operation & Procedures can be anything from clearing an ear canal to a full lung transplant. Separating the two extremes cannot be achieved simply by truncating the hierarchy at a set byte level. Instead, specific codes that were deemed relevant were selected at 2-byte level and their potential severity was classified as (High {H}, Medium {M}, Low {L}) depending on the potential procedures impacting an individual's health or their ability to be active.

- Nervous system operations (70***) {H}
- Endocrine system and breast operations (71***) {H}
- Eye Operations (72***) {M}
- Ear operations (73***) {L}
- Respiratory tract operations (74***) {H}
- Mouth Operations (75***) {M}

- Upper digestive tract operations (76***) {M}
- Lower digestive tract operations (77***) {H}
- Other abdominal organ operations (78***) {H}
- Heart Operations (79***) {H}
- Artery and Vein Operations (7A***) {H}
- Urinary operations (7B***) {M}
- Male genital organ operations (7C***) {H}
- Lower female genital tract operations (7D***) {H}
- Upper female genital tract operations (7E***) {H}
- Obstetric operations/Pregnancy operations (7F***) {H}
- Skin Operations (7G***) {M}
- Soft Tissue operation (7H***) {H}
- Skull or spine, bone and joint operations (7J***) {H}
- Other bone and joint operations (7K***) {H}
- Miscellaneous operations (7L***) {H}
- Diagnostic imaging, testing and rehabilitation (7P***) {L}

A – Infection and Parasitic Diseases

This is the diagnostic chapter for infectious and parasitic diseases like Syphilis, with a 46.38% prevalence and average occurrence of 2.25. This is a diagnostic chapter, and all subchapters were included at a 2-byte depth.

B – Neoplasms (Cancers)

This chapter includes different types of cancer-related diagnoses, with 36.3% prevalence and occurrence 2.07. These codes were considered at 2-byte depth as it is possible to differentiate between cancerous and benign neoplasms at this level. Cancers, including treatment procedures such chemotherapy, are known to have a severe effect on an individual's mental and physical wellbeing which could affect their activity and sleep levels.

C – Endocrine, nutritional, metabolic and immunity disorders

This is a large and diverse chapter holding large amounts of codes for diabetes and vitamin deficiencies, which appears in 33.29% of individuals at an (average occurrence 3.41). This chapter is extremely technical and granular, most of the key differentials are done at 5-byte depth, such as differentiating between complications of Type-1 and Type-2 diabetes. This chapter was taken at a 3-byte depth with the understanding that these subchapters are still diverse and more granular.

D – Diseases of blood and blood-forming organs

This contains conditions like Anaemia, with 7.44% prevalence. All subchapters were taken at a 2-byte depth.

E – Mental disorders

Conditions such as Psychosis or Learning Disabilities, 28.38% prevalence, average occurrence 2.83. All subchapters were taken at a 2-bytes depth.

F – Nervous system and sense organ diseases

Chapter holds information on Meningitis related conditions and sense organ diseases such as Deafness (62.71% prevalence, occurrence 3.46). All subchapters were taken at a 2-bytes depth.

G – Circulatory system diseases (Cardiac/Heart Diseases)

Holds codes relating to the Heart or Circulation of Blood (57.15% prevalence, occurrence 4.67). All subchapters were taken at a 2-bytes depth.

H – Respiratory system diseases

Holds conditions such as Pneumonia and Lung Diseases, 58.12% prevalence, average occurrence of 3.81. All subchapters were taken at a 2-bytes depth.

J – Digestive system diseases

Holds conditions relating to the Mouth, Stomach and Lower Digestive Tract, 45.81% prevalence, average occurrence of 2.76. All subchapters were taken at a 2-bytes depth.

K – Genitourinary system diseases

Holds conditions relating to the Genital and Urinary Glands, Table 1 shows this exists in 50.86% of individuals at an average occurrence of 3.14. Most codes found were for UTI's and conditions relating to the female anatomy. All subchapters were taken at a 2-bytes depth as it is possible to differentiate between male, female and common codes.

M – Skin and subcutaneous tissue diseases

Describes the conditions such as Sore Skin or Ulcers, 64.28% prevalence at an average occurrence of 3.49. The codes in the chapter are mostly mild so this chapter was accepted at a 1-byte depth.

N – Musculoskeletal and connective tissue diseases

Holds conditions such as Arthritis, 73.67% prevalence at an average occurrence of 4.89. All subchapters were taken at a 2-bytes depth.

S – Injury and poisoning

Holds codes for injuries such as Broken Bones and Overdoses, 52.61% prevalence,. average occurrence of 2.51. This chapter has a large range of severity between the codes, from Fractured Skull to Wasp Sting. All subchapters were taken at a 2-bytes depth as then it is possible to differentiate between the severity of the subchapters at this level.

Two additional chapters: 2 – Examinations / Signs, and 4 – Laboratory Procedures, were excluded on the basis of their very high prevalence (98.42%, 97.22%) and thus low selectivity.

Table 2 shows the impact of removing and truncating chapters to a set byte-depth as described above. There is a small reduction in the number of individuals, this is to be expected. There is also a drastic reduction in the number of events, this is partially down to the amount of information deemed not useful, but this is mostly down to the exclusion of chapters 2 & 4 which have the highest number of occurrences. Most notably, we have reduced the number of different codes from 28,109 to 215. This is a significant reduction in the amount of information needed to process.

	**CPH Population Full**	**CPH Population Truncated**	**% Change**
**Individuals**	19,852	19,617	-1.18%
**Events**	10,133,630	702,955	-93.06%
**Unique Codes**	28,109	215	-99.24%

Table 2: Analysis of truncating Read codes for GP Clinical

Assignment of Health Impact Scores

Assigning a code impact score

The scores assigned to the events in the chapters listed above reflect the criteria that minor events will occur more frequently than major events. A tiered system was used to numerically classify the event based on impact to health, High {100}, Medium {10} and Low {1}. The average of these scores is then used to normalise the effects on individuals having larger numbers of events from High Impacts chapters. With the higher the value, the less likely they are to be considered physically active.

Note that individuals who have no events recorded within the specified time frame are assigned 0 health impact score and we will assume their activity levels are not impaired.

Table 3 shows the impact of using codes surrounding individual's activity tracker readings (6 months before and 1 month after). As expected, there is a reduction of individuals and events, as well as a reduction in unique codes, meaning I could have assigned impacts scores to 171 codes instead of 215.

	CPH Population Truncated	Events Surrounding Activity Tracker	% Change
**Individuals**	19,617	8,833	-54.97%
**events**	702,955	27,737	-96.1%
**Unique Codes**	215	171	-20.47%

Table 3: Analysis of taking events surrounding individual's activity tracker readings

Table 4 shows the distribution of individuals over score ranges. The distribution is what was expected, around 75% of individuals do not have an event with a High impact score.

Individuals Health Impact Score Range	Number of individuals in range
**0**	11,019 (55.5%)
**>0 to <=10**	3,468 (17.5%)
**>10 to <=50**	2,010 (10.1%)
**>50**	3,355 (16.9%)

Table 4: Health Impact Score Ranges
