# Supplementary material for: Using Wearable Activity Trackers to Predict Type 2 Diabetes: Machine Learning–Based Cross-sectional Study of the UK Biobank Accelerometer Cohort
Source: JMIR Diabetes. 2021 Mar 19;6(1):e23364. doi: 10.2196/23364 (PMC8080299; doi:10.2196/23364)
Supplement: Multimedia Appendix 2 [file diabetes_v6i1e23364_app2.pdf]

Unsupervised Clustering Analysis

Clustering analysis was performed to assess whether it is possible to achieve a natural and meaningful grouping of participants based on their high-level activity bout features. Firstly, clustering algorithms were used to partition participants based on similarity in their activity patterns, regardless of whether they have been labelled as T2D or control.

Secondly, in order to interpret the clusters, further analysis was performed to determine how the T2D positive outcome is distributed across clusters, and to test for statistically significant differences in the distribution of socio-demographic and lifestyle variables across different clusters. These can be taken as an indication that differences in physical activity signals do provide a significant segregation of participants, reinforcing a known hypothesis This analysis method has been adopted in other studies, for instance to identify groups of adolescents at risk of developing chronic disease and also in the UK Biobank to cluster overweight and obese adults.

Both *k*-means and agglomerative hierarchical clustering were used to achieve step 1. *k*- means often produces stronger, cohesive clusters, however it requires that the optimal value for the *k* hyperparameter to be identified. This was done using the *elbow* method, which selects the optimal number of clusters by fitting the *k* means models on a range of values for *k* and plots it in a line chart, as in Figure 1 where the Silhouette Score is used as a measure of cluster quality. The *elbow* point of inflection on this curve indicates the model fits best at *k=4*.

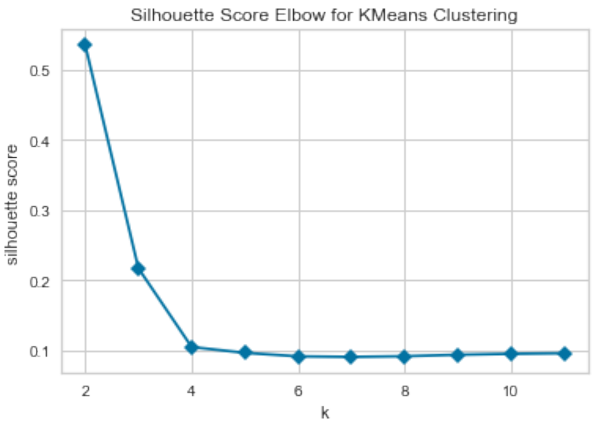

Figure 1 Elbow curve for k-means. Point of inflection shows 4 is optimal value for k

In contrast, hierarchical clustering adopts a *bottom-up* approach where each observation starts in its own cluster, and pairs of clusters are merged as one moves up the hierarchy. The Ward function was used as the linkage function which minimises the sum of squared differences within all clusters. This function shows that at a cutoff distance of 300, there three distinct clusters, as seen in the dendrogram in Figure 2. We determine that these are the three clusters to be used for this clustering method, and 4 for *k*-means.

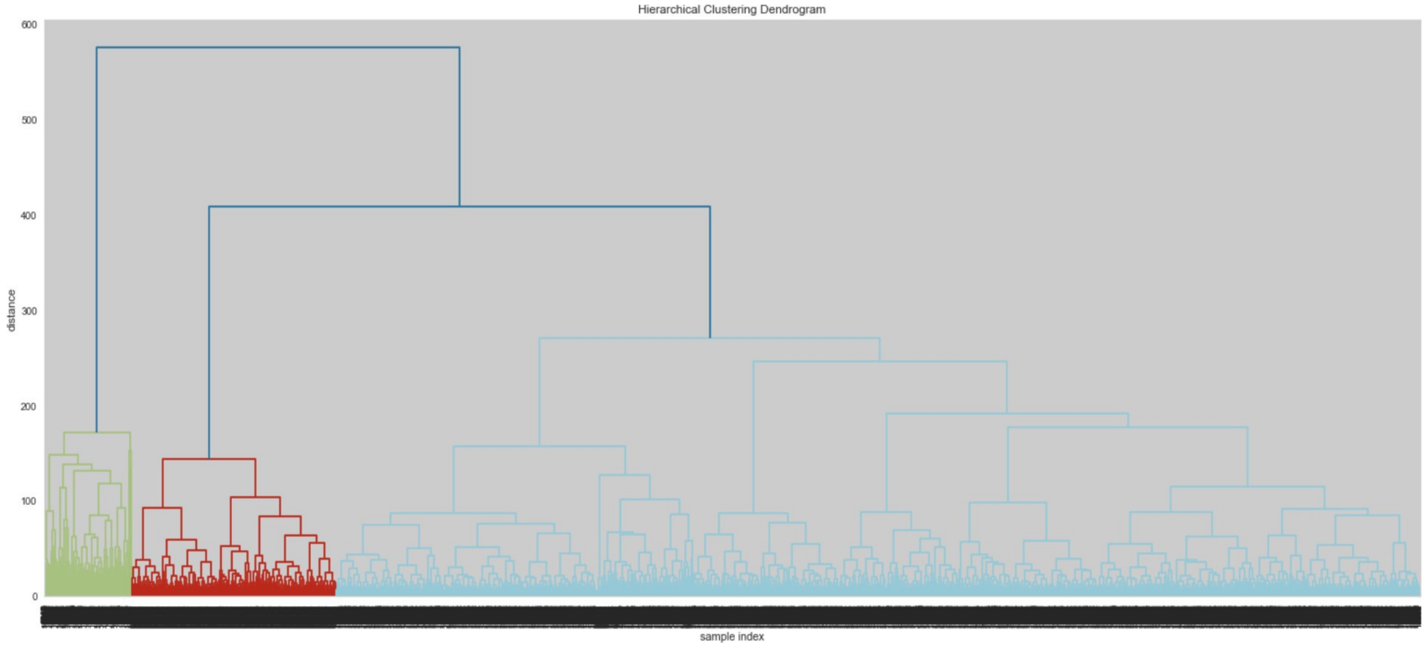

Figure 2 dendrogram visualisation of three distinct clusters identifiable at distance threshold 300.

Results

Clustering Results

T-SNE for dimensionality reduction to produce a 2D rendering of both k-means and hierarchical clusters, shown in Figure 3 and Figure 4respectively. Different colors indicate different clusters. The clusters are much better defined using hierarchical clustering than for *k*- means, where there is significant qualitative overlap, with poor separation for dimensionality reduction to produce a 2D rendering of both k-means and hierarchical clusters, shown in Figure 3 and Figure 4 respectively. Different colors indicate different clusters. The clusters are much better defined using hierarchical clustering than for *k*-means, where there is significant qualitative overlap, with poor separation.

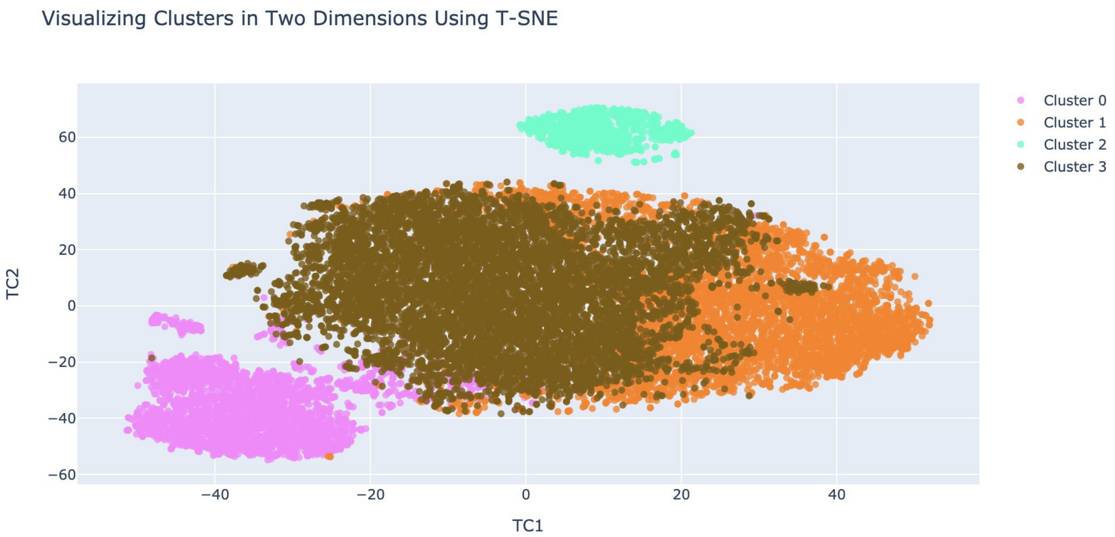

Figure 3 T-SNE visualisation for k-means clustering. 4 closely overlapping clusters can be seen.

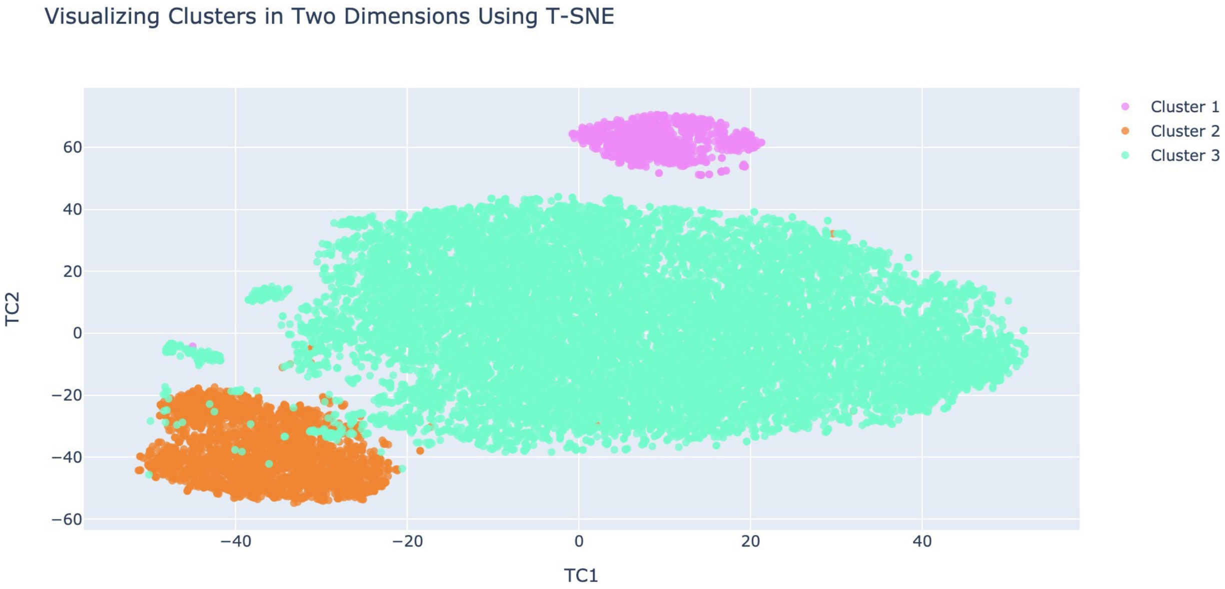

Figure 4 T-SNE visualisation for hierarchical clustering. 3 cohesive and separated clusters can be seen.

This observation has been quantified using Silhouette coefficients and the Calinski-Harabrasz scores.
Silhouette measures similarity of observations within each cluster (cohesion) compared to those in other clusters (separation). Values range from -1 to +1, where a higher value implies the observation is more matched to its own cluster and less so to neighbouring clusters.

The Calinski-Harabrasz metric is well-suited where the ground truth is not known. It measures the ratio of the sum of intra-cluster dispersion and of inter-cluster dispersion for all clusters. Dispersion is defined as the squared sum of distances between any two points in a cluster. There is no cut-off value for this performance metric as it is used for comparison between different clustering methods. A higher Calinski-Harabrasz score implies better defined clusters. Values for these metrics are reported in Table 1 showing that hierarchical clustering produced better defined and cohesive clusters than *k*-means. So, only the results of this algorithm were further analyzed.

	Calinski-Harabrasz	Silhouette
***k*-means**	2390.53	0.105
**Hierarchical**	2720.14	0.207

Table 1 Calinski-Harabrasz and Silhouette scores for unsupervised, resulting clusters by high-level activity bout features using k-means and hierarchical clustering algorithms

The fraction of males, 1,884 out of 2,936 (64.2%), is higher in Cluster 2 than in the other two clusters, as are the number of individuals in the highest age bracket (60 to 70). This trend is seen in most of the other socio-demographic and lifestyle features which are associated with exhibiting poorer health. Most significantly, the fraction of each cluster that is T2D positive was found to be 2,930 out 2,936 (99.8 %) in Cluster 2, with much smaller distributions in Clusters 1 and 3. This is also reflected in the broader context of the other socio-demographic and lifestyle features, where phenotypes associated with increased risk of T2D (increasing age, high body fat percentage and a sedentary lifestyle) are also highly expressed in Cluster 2.

The chi-square tests in *p*-value columns in also show a significant correlation between most of the features and Cluster membership, in particular those relating to anthropometric measures such as age, sex, body fat percentage and waist circumference.

Table 2 provides an interpretation of clusters in terms of socio-demographic and lifestyle features, showing a non-random distribution of the variables within each cluster in all cases. Again Cluster 2 stands out.

Note that numerical variables such as waist circumference were discretized into categorical features.

		Cluster 1	Cluster 2	Cluster 3	*p*-value
**Cluster size**		1,119	2,936	13,306	
**Number of T2D positive**		162	2,930	339	*P* <.001
**Number of Males**		564	1884	6107	*P* <.001
**Age (n)**					*P* <.001
	40 to 49	249	255	2697	
	50 to 59	411	854	4583	
	60 to 70	459	1826	6029	
**Body fat percentage (Male)**					*P* <.001
	6 % to 13%	23	13	347	
	14% to 17%	56	54	817	
	18% to 25%	434	609	5487	
	Over 25%	607	2260	6654	
**Body fat percentage (Female)**					*P* <.001
	10% to 12%	2	0	5	

	14% to 20%	13	6	169	
	21% to 24%	36	23	467	
	25% to 31%	210	116	2692	
	Over 31%	857	2791	9973	
**Smoking status**					*P* <.001
	Never	607	1332	7706	
	Past	416	1377	4749	
	Current	95	227	852	
**Alcohol consumption status**					*P* <.001
	Never	36	132	357	
	Past	29	132	446	
	Current	1054	2672	12637	
**Ethnic group**					*P* <.001
	White	1101	2866	13214	
	Mixed	4	5	25	
	Asian	0	15	16	
	Black African	4	18	33	
	Chinese	1	10	17	
	Other	8	22	64	
**Time spent watching television**					*P* <.001
	Less than one hour	275	390	3158	
	1 to 2 hours	330	709	3860	
	2 to 3 hours	234	1065	3147	
	Over 3 hours	279	772	3143	
**Sleep duration**					*P* <.001
	Less than 7 hours	238	775	2883	
	7 to 8 hours	810	1873	9632	
	Over 8 hours	71	287	790	
**Waist circumference (Male)**					*P* <.001
	Less than 94cm	504	617	6612	
	94cm to 102cm	265	667	3307	
	Over 102cm	350	1652	3388	
**Waist circumference (Female)**					*P* <.001
	Less than 80cm	507	289	6390	
	80 to 88 cm	271	415	3136	
	Over 88cm	341	2232	3780	

**Duration of walking activity**					*P*= .37
	0 to 20 mins	361	1035	4533	
	21 to 30 mins	272	694	3168	
	31 to 60 mins	329	864	3884	
	Over 60 mins	157	343	1720	
**Duration of moderate activity**					*P* <.001
	0 to 15 mins	211	688	2484	
	16 to 30 mins	403	997	4765	
	31 to 60 mins	337	822	4139	
	61 to 180 mins	168	428	1917	
**Duration of vigorous activity**					*P* <.001
	0	9	17	94	
	1 to 20 mins	360	1267	4511	
	21 to 45 mins	410	896	4430	
	46 to 180 mins	341	757	4270	

Table 2 Distribution of discretized sociodemographic, lifestyle and anthropometry characteristics across the three resulting clusters. P-values are also shown at the right-most column.

In Figures 5-7 we can see that the daily relative proportions of time spent in each activity type for each cluster does not vary greatly between different clusters. But if these physical activity features include the average number of bouts and length of bouts for activity, then it can be seen there are differences in each cluster.

The example for sleep activity shown in Figures 8-10**.** and Figure 10 demonstrate different aggregated distributions for the average daily number of sleep bouts, daily average length of sleep bouts, and average daily percentage time spent sleeping. This trend is seen in the other activity types. With further granularization, these differences become more pronounced.

The combination of the granularized patterns of physical activity bout features produce significant differences that enable these individuals to be segregated into well-defined groups.

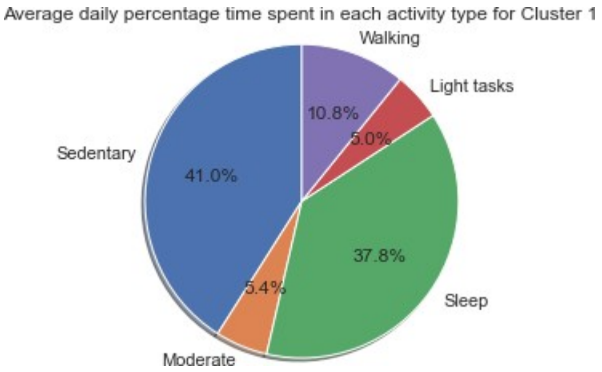

Figure 5 Average daily percentage time spent in each activity type for Cluster 1

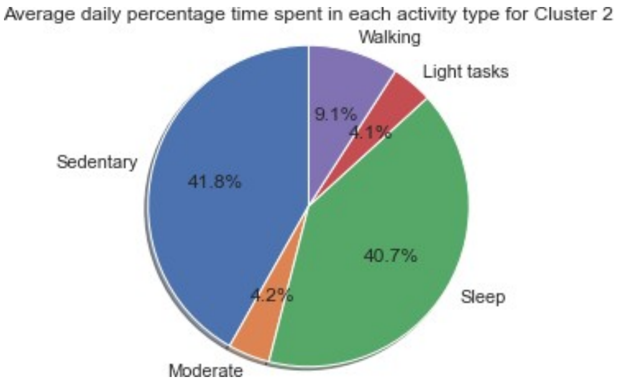

Figure 6 Average daily percentage time spent for each activity type, for Cluster 2

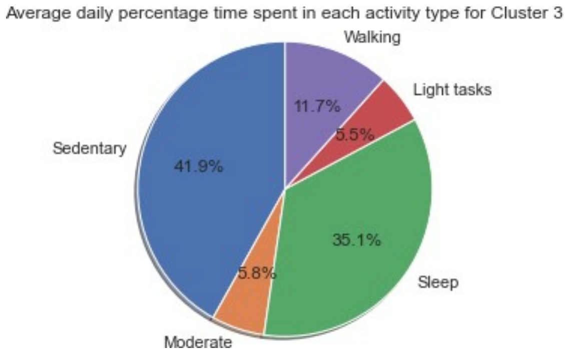

Figure 7 Average daily percentage time spent for each activity type, for Cluster 3

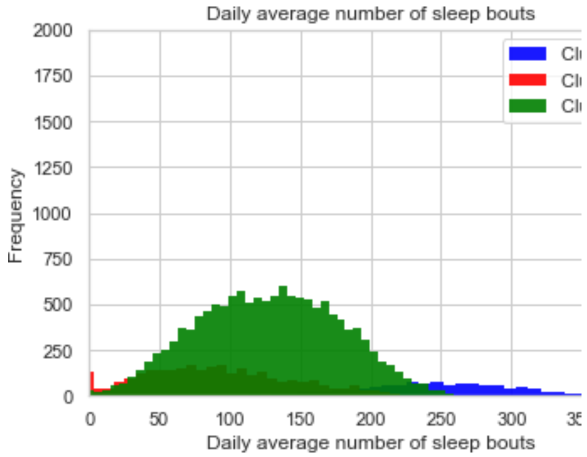

Figure 8 Daily average number of sleep bouts for all clusters

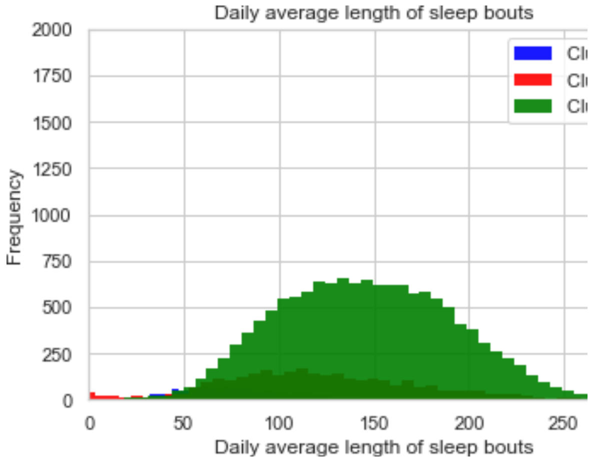

Figure 9 Daily average length of sleep bouts for all clusters

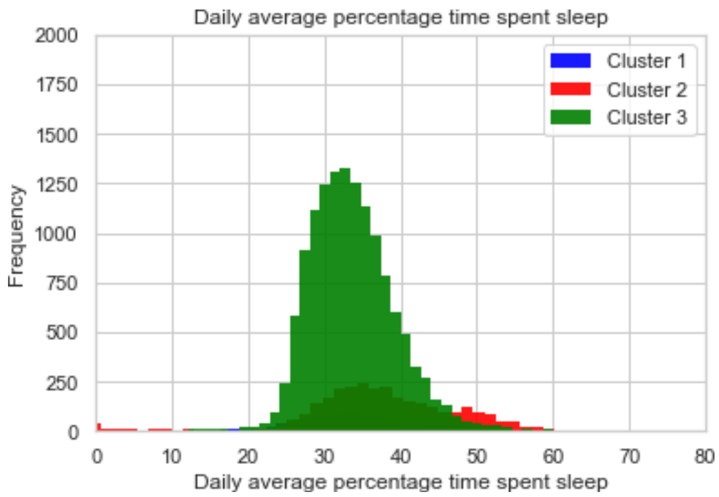

Figure 10 Daily average percentage time spent in sleep for all clusters
