# Supplementary material for: Using Wearable Activity Trackers to Predict Type 2 Diabetes: Machine Learning–Based Cross-sectional Study of the UK Biobank Accelerometer Cohort
Source: JMIR Diabetes. 2021 Mar 19;6(1):e23364. doi: 10.2196/23364 (PMC8080299; doi:10.2196/23364)

<u>Multimedia Appendix B: T2D prediction results from a suite of machine learning models</u>

This appendix displays all the performance metrics results for the predictive models described in the paper. Altogether, 18 models were built for the prediction of T2D from different training sets. Each model used a different classifier (either random forest, logistic regression or XGBoostt), a different feature set (either HLAF and SDL combined, HLAF exclusively, or SDL exclusively) and a different negative control population (either highly impaired in terms of physical activity or without physical activity impairment according to the severity scores in **Multimedia Appendix A.** All models shown underwent 10-fold cross-validation for robustness.

There are two tables associated with each model: A confusion matrix for the model's predictions and a table to display standard machine learning model performance metrics: Precision, recall and F1-score.

Additionally, for each model, the ROC curve is displayed with the AUC scores of each separate model in the 10 folds displayed. This is more detailed than what it seen in the paper.

Section 1 displays the results for the models that use the T2D positive population against the normoglycaemic controls without high levels of physical activity impairment, *Norm-0*, where the impairment severity score is equal to 0.

Section 2 displays the results that use the T2D positive population against the normoglycaemic controls with high levels of physical activity impairment, *Norm-2*, where the impairment severity score is 50 or above.

Section 1: T2D vs *Norm-0*

HLAF + SDL combined

Random forest:

Confusion Matrix

	T2D Negative	T2D Positive
T2D Negative	3491	687
T2D Positive	917	2186

	Precision	Recall	F1-score
T2D Negative	0.79	0.84	0.81
T2D Positive	0.76	0.70	0.73

AUROC scores and ROC curve

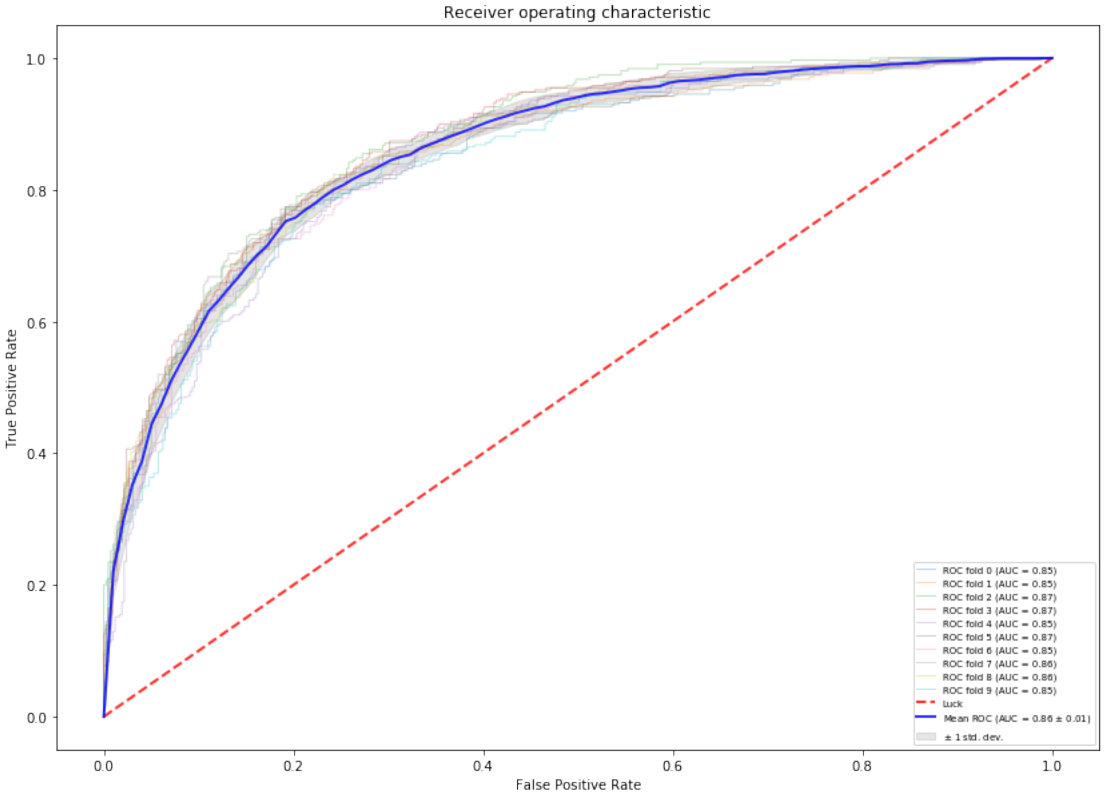

Logistic regression

Confusion Matrix

	T2D Negative	T2D Positive
T2D Negative	3520	658
T2D Positive	896	2207

	Precision	Recall	F1-score
T2D Negative	0.80	0.84	0.82
T2D Positive	0.77	0.71	0.74

AUROC scores and ROC curve

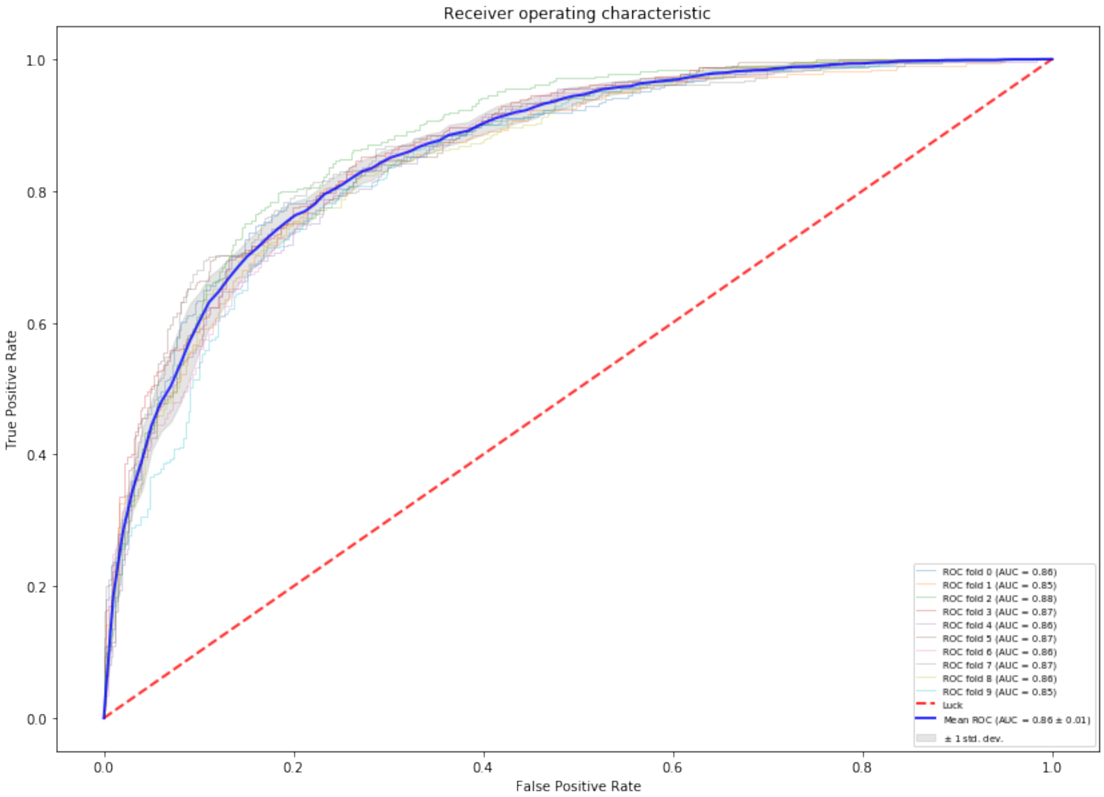

XGBoost

Confusion Matrix

	T2D Negative	T2D Positive
T2D Negative	3390	788
T2D Positive	874	2229

	Precision	Recall	F1-score
T2D Negative	0.80	0.81	0.80
T2D Positive	0.74	0.72	0.73

AUROC scores and ROC curve

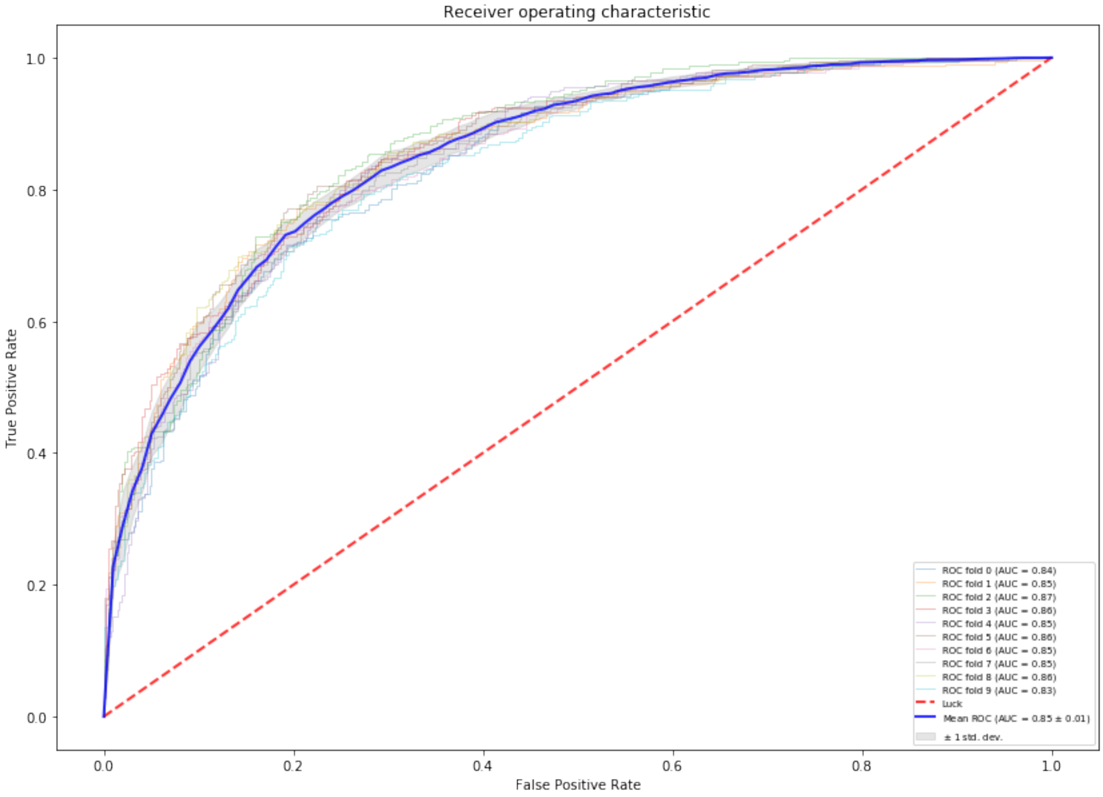

[HLAF Exclusive]

Random forest:

Confusion Matrix

	T2D Negative	T2D Positive
T2D Negative	3502	676
T2D Positive	1275	1828

	Precision	Recall	F1-score
T2D Negative	0.73	0.84	0.78
T2D Positive	0.73	0.59	0.65

AUROC scores and ROC curve

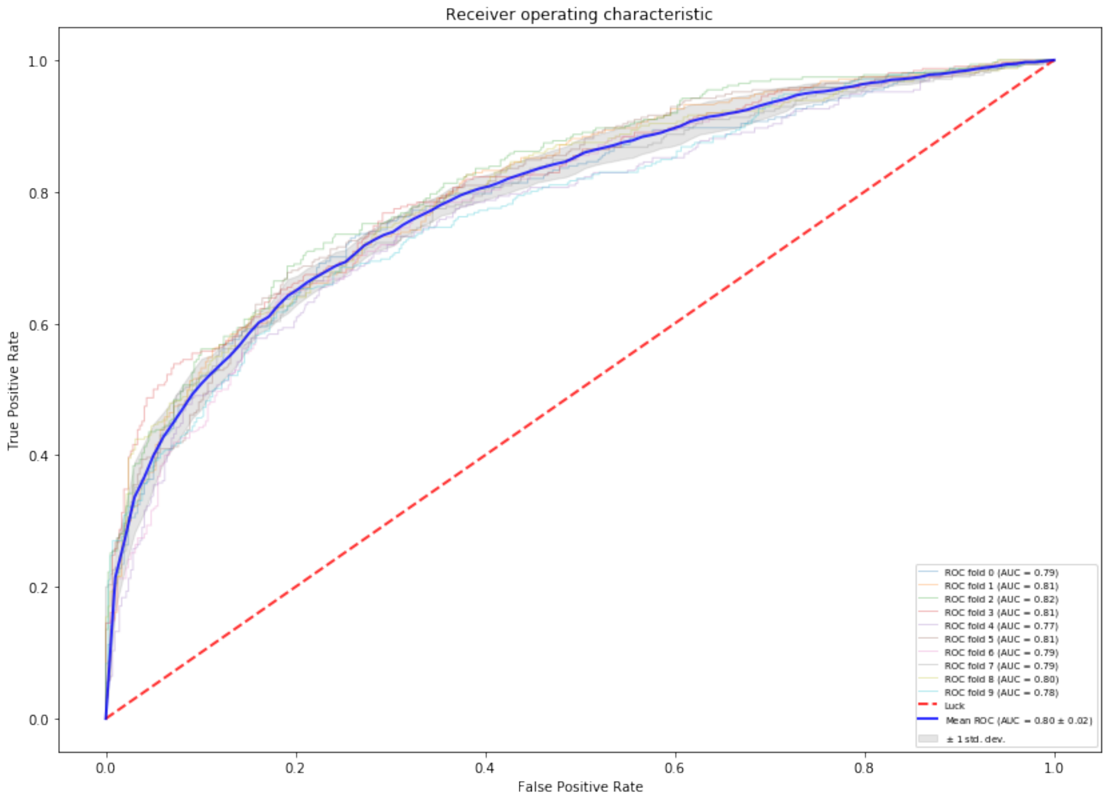

Logistic regression

Confusion Matrix

	T2D Negative	T2D Positive
T2D Negative	3377	801
T2D Positive	1162	1941

	Precision	Recall	F1-score
T2D Negative	0.74	0.82	0.77
T2D Positive	0.71	0.63	0.66

AUROC scores and ROC curve

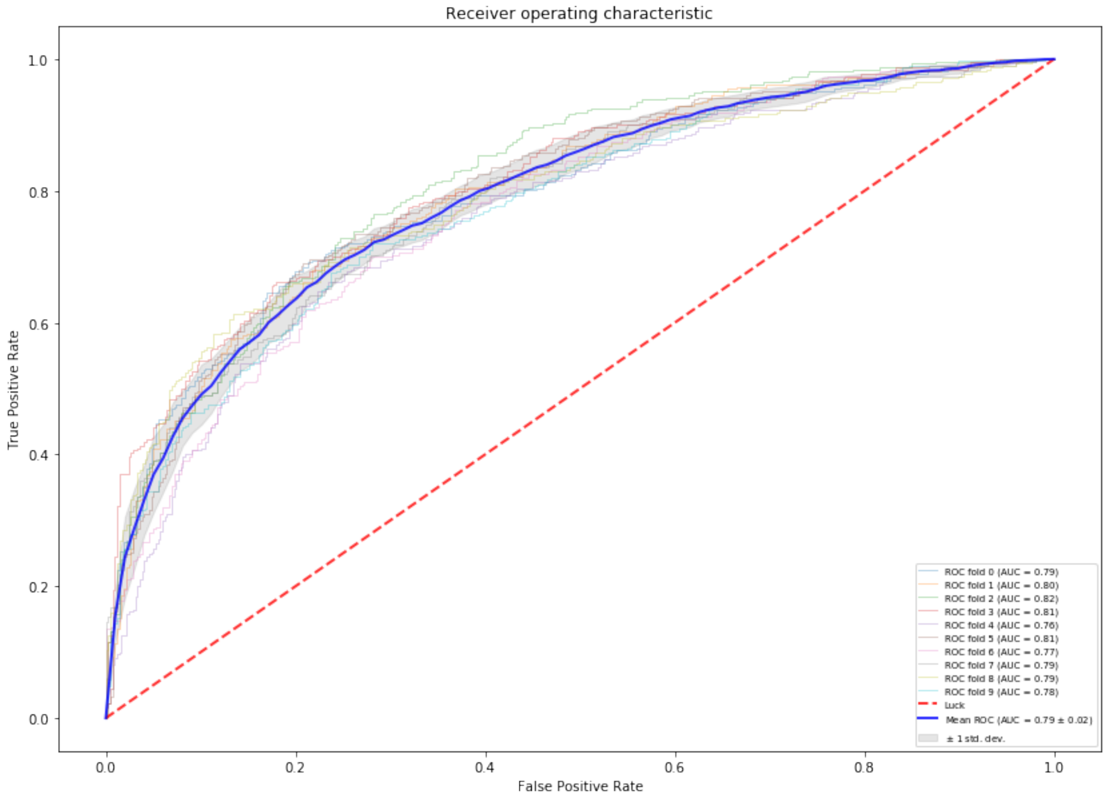

XGBoost

Confusion Matrix

	T2D Negative	T2D Positive
T2D Negative	3371	807
T2D Positive	1197	1906

	Precision	Recall	F1-score
T2D Negative	0.74	0.81	0.77
T2D Positive	0.70	0.61	0.66

AUROC scores and ROC curve

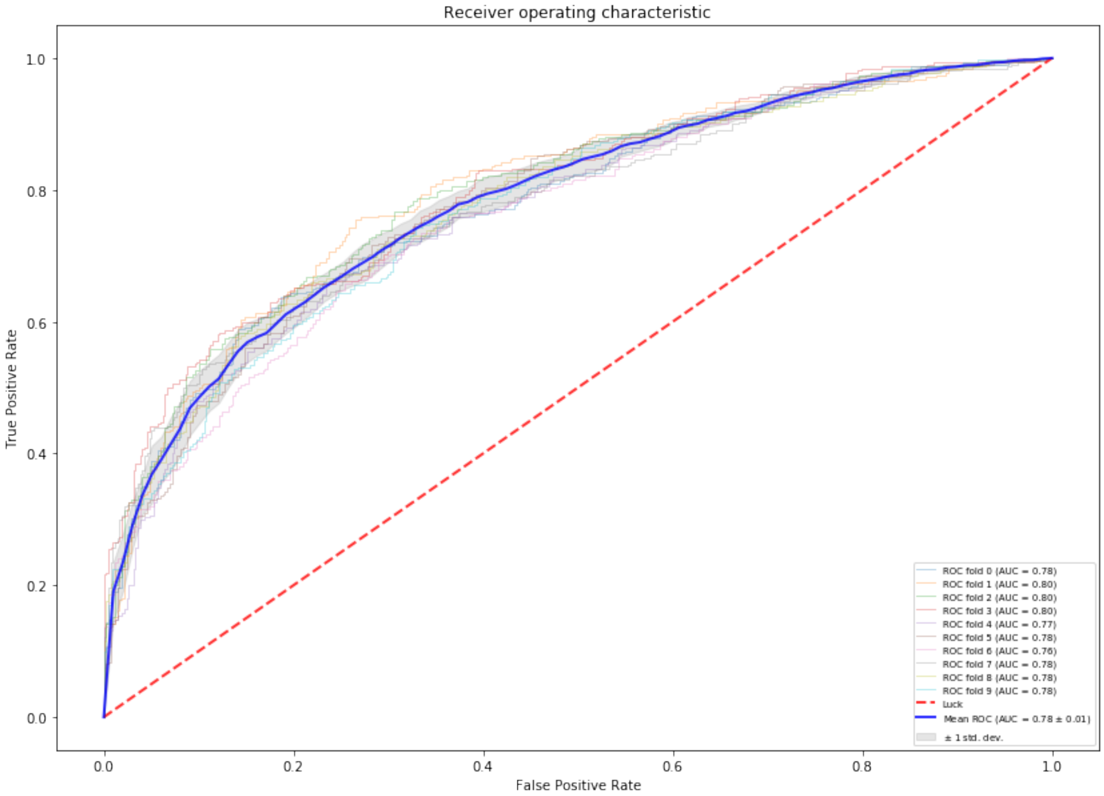

<u>SDL Exclusive</u>

Random forest:

Confusion Matrix

	T2D Negative	T2D Positive
T2D Negative	3260	918
T2D Positive	971	2132

	Precision	Recall	F1-score
T2D Negative	0.77	0.78	0.78
T2D Positive	0.70	0.69	0.69

AUROC scores and ROC curve

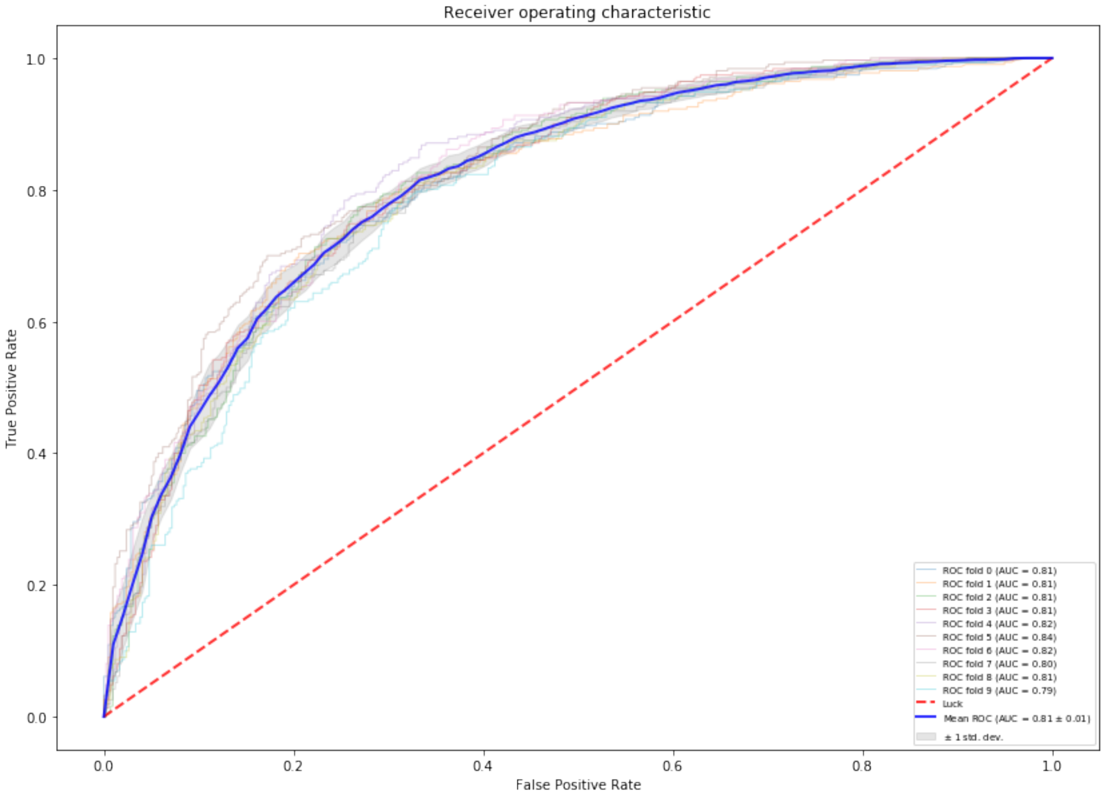

Logistic regression

Confusion Matrix

	T2D Negative	T2D Positive
T2D Negative	3379	7899
T2D Positive	1040	2063

	Precision	Recall	F1-score
T2D Negative	0.76	0.81	0.79
T2D Positive	0.72	0.66	0.69

AUROC scores and ROC curve

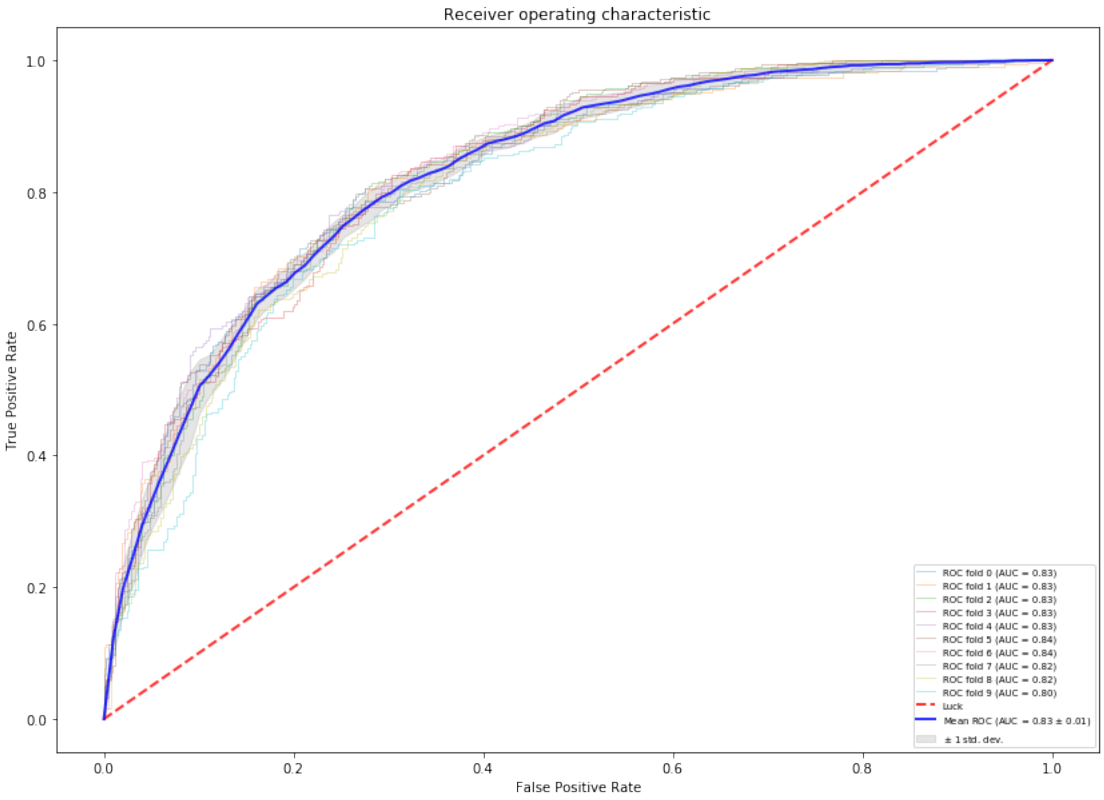

XGBoost

Confusion Matrix

	T2D Negative	T2D Positive
T2D Negative	3199	979
T2D Positive	1039	2064

	Precision	Recall	F1-score
T2D Negative	0.75	0.77	0.76
T2D Positive	0.68	0.67	0.67

AUROC scores and ROC curve

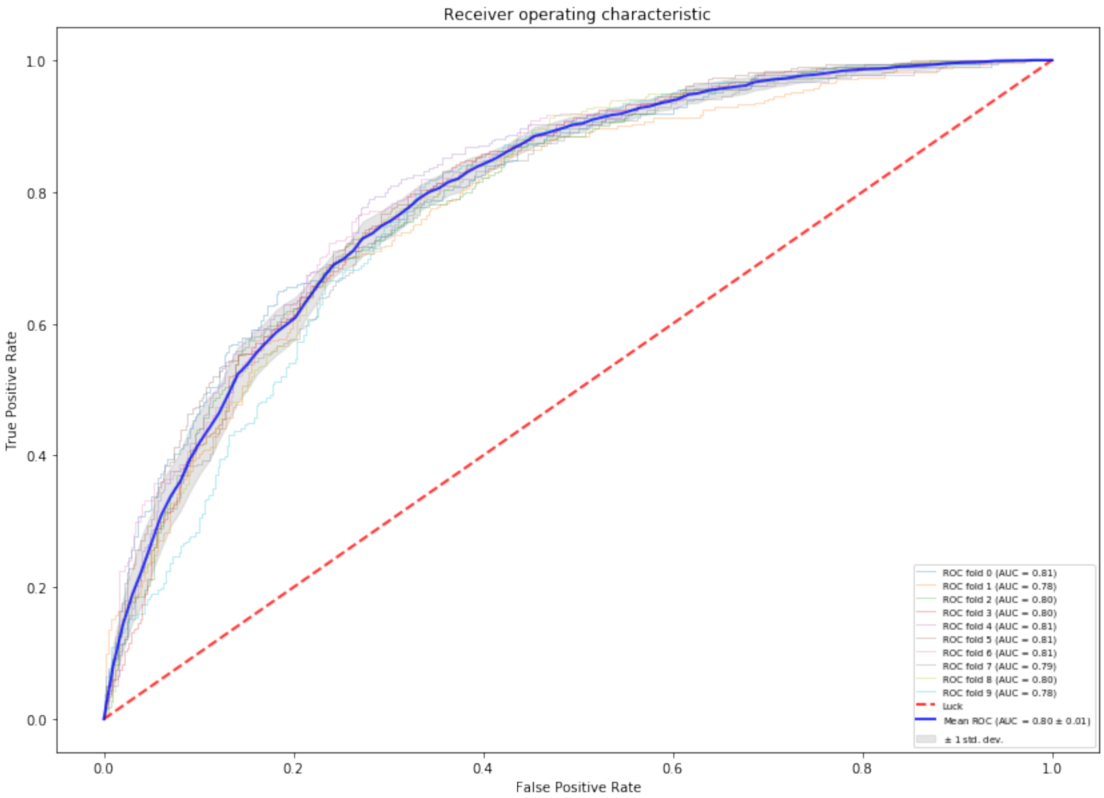

Section 2: T2D vs *Norm-2*

HLAF + SDL combined

Random forest:

Confusion Matrix

	T2D Negative	T2D Positive
T2D Negative	936	730
T2D Positive	372	1862

	Precision	Recall	F1-score
T2D Negative	0.72	0.56	0.63
T2D Positive	0.72	0.83	0.77

AUROC scores and ROC curve

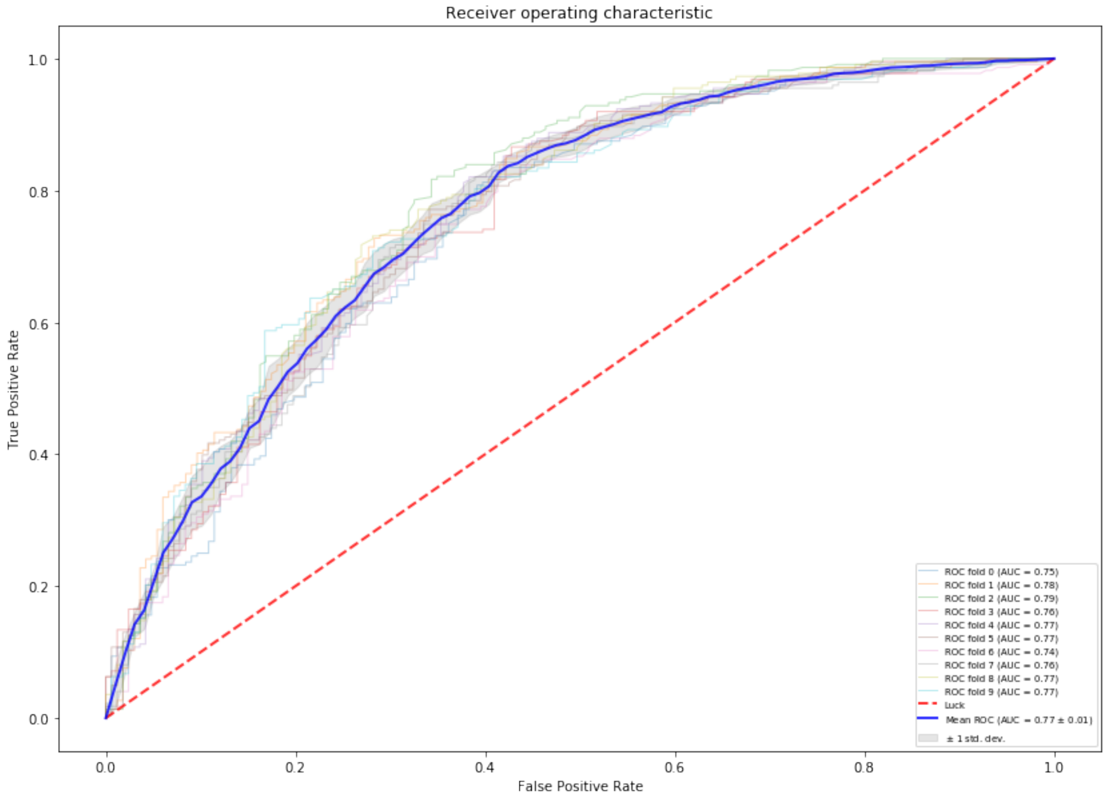

Logistic regression

Confusion Matrix

	T2D Negative	T2D Positive
T2D Negative	1024	642
T2D Positive	447	1787

	Precision	Recall	F1-score
T2D Negative	0.70	0.61	0.65
T2D Positive	0.74	0.80	0.77

AUROC scores and ROC curve

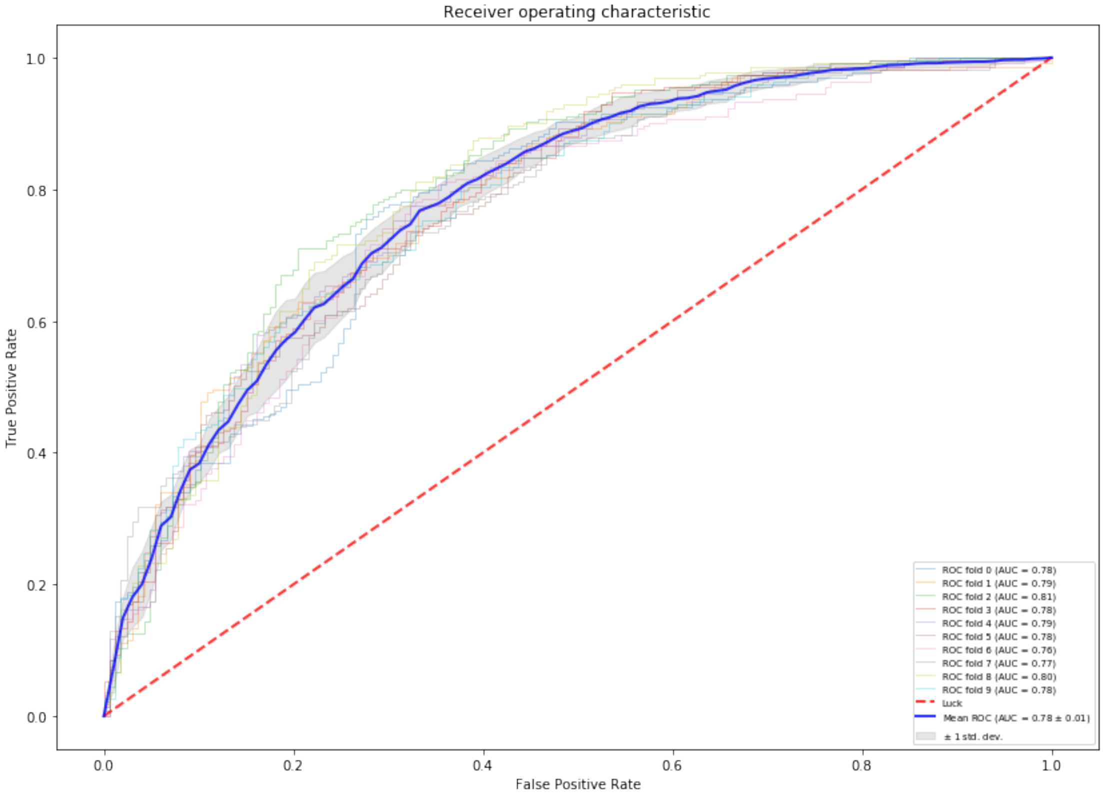

XGBoost

Confusion Matrix

	T2D Negative	T2D Positive
T2D Negative	966	700
T2D Positive	447	1787

	Precision	Recall	F1-score
T2D Negative	0.68	0.58	0.63
T2D Positive	0.72	0.80	0.76

AUROC scores and ROC curve

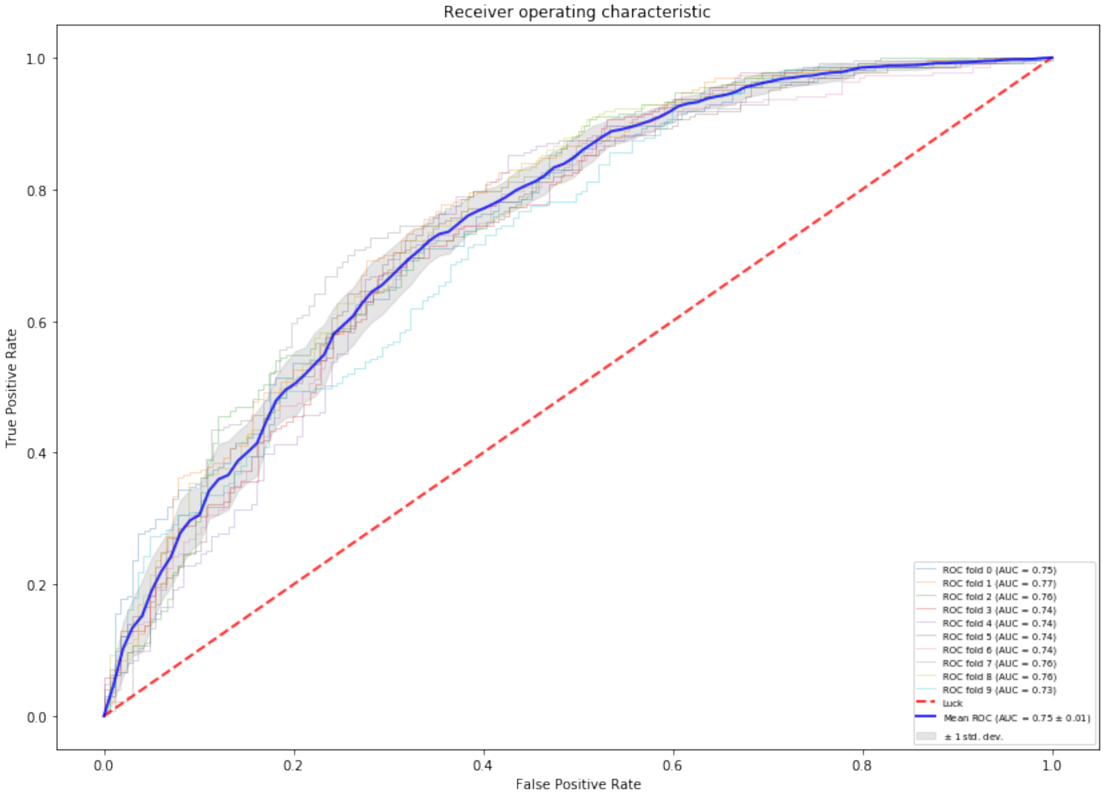

HLAF Exclusive

Random forest:

Confusion Matrix

	T2D Negative	T2D Positive
T2D Negative	810	856
T2D Positive	552	1682

	Precision	Recall	F1-score
T2D Negative	0.59	0.49	0.54
T2D Positive	0.66	0.75	0.70

AUROC scores and ROC curve

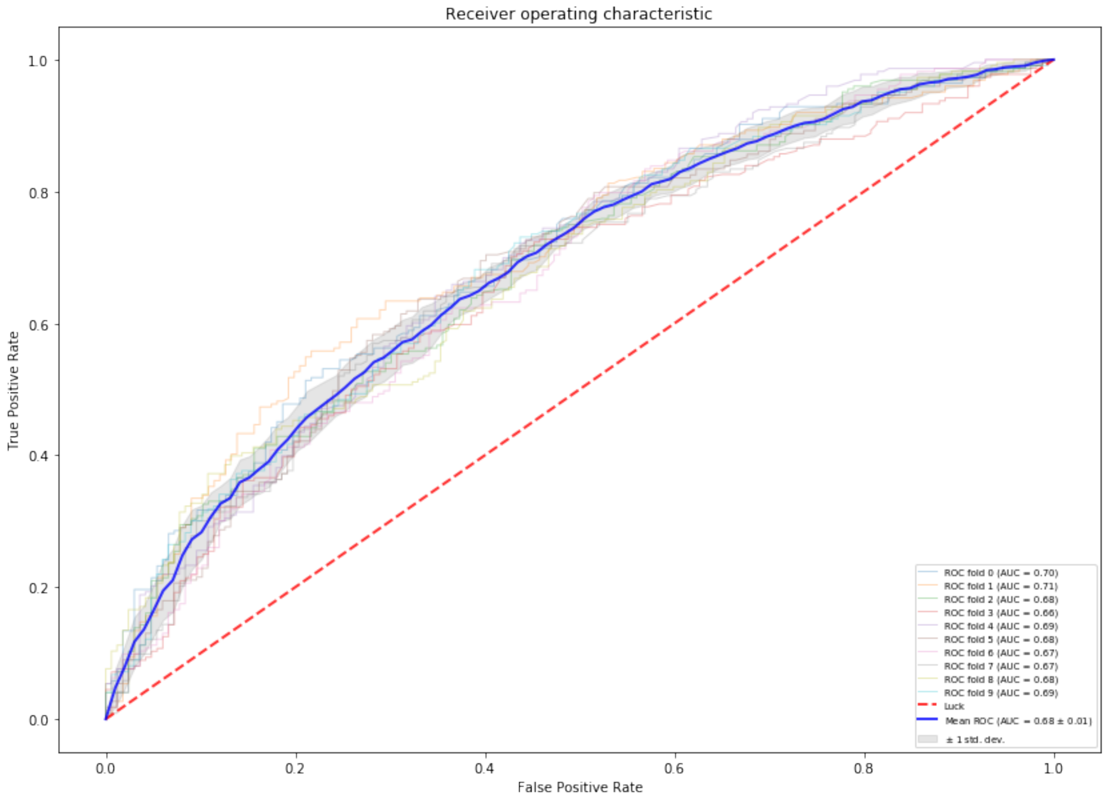

Logistic regression

Confusion Matrix

	T2D Negative	T2D Positive
T2D Negative	796	870
T2D Positive	502	1732

	Precision	Recall	F1-score
T2D Negative	0.61	0.48	0.54
T2D Positive	0.67	0.78	0.72

AUROC scores and ROC curve

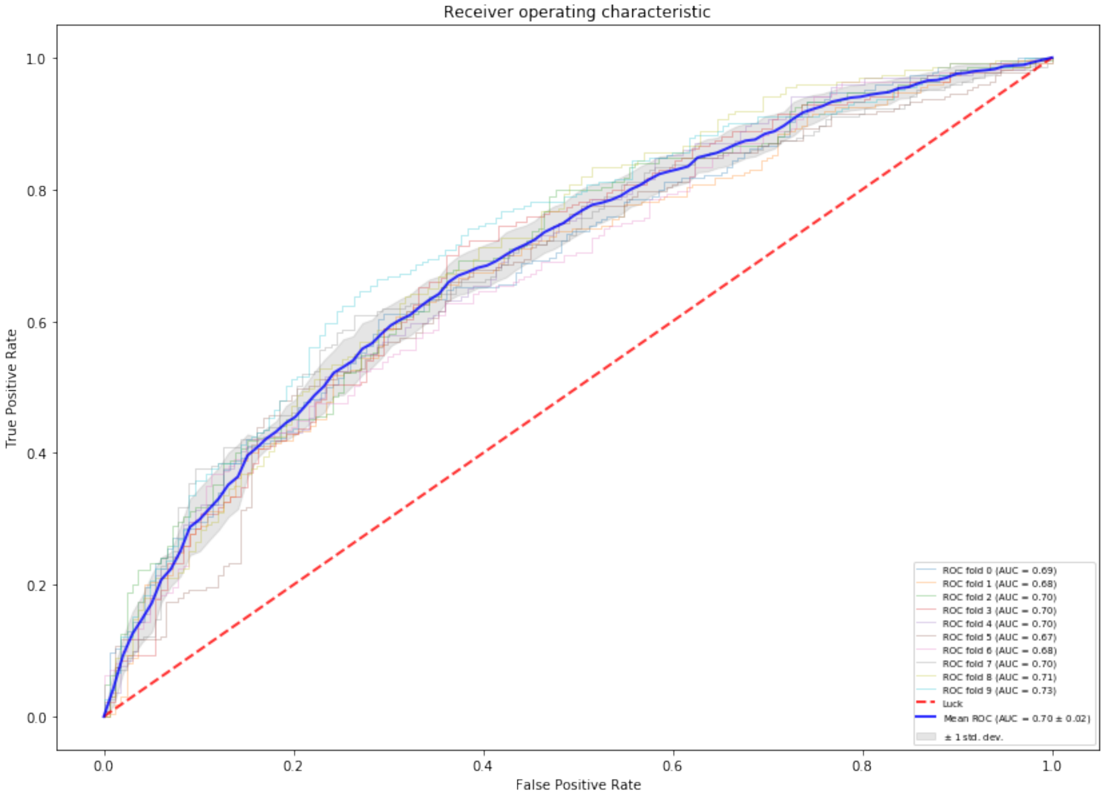

XGBoost

Confusion Matrix

	T2D Negative	T2D Positive
T2D Negative	813	853
T2D Positive	657	1577

	Precision	Recall	F1-score
T2D Negative	0.55	0.49	0.52
T2D Positive	0.65	0.71	0.68

AUROC scores and ROC curve

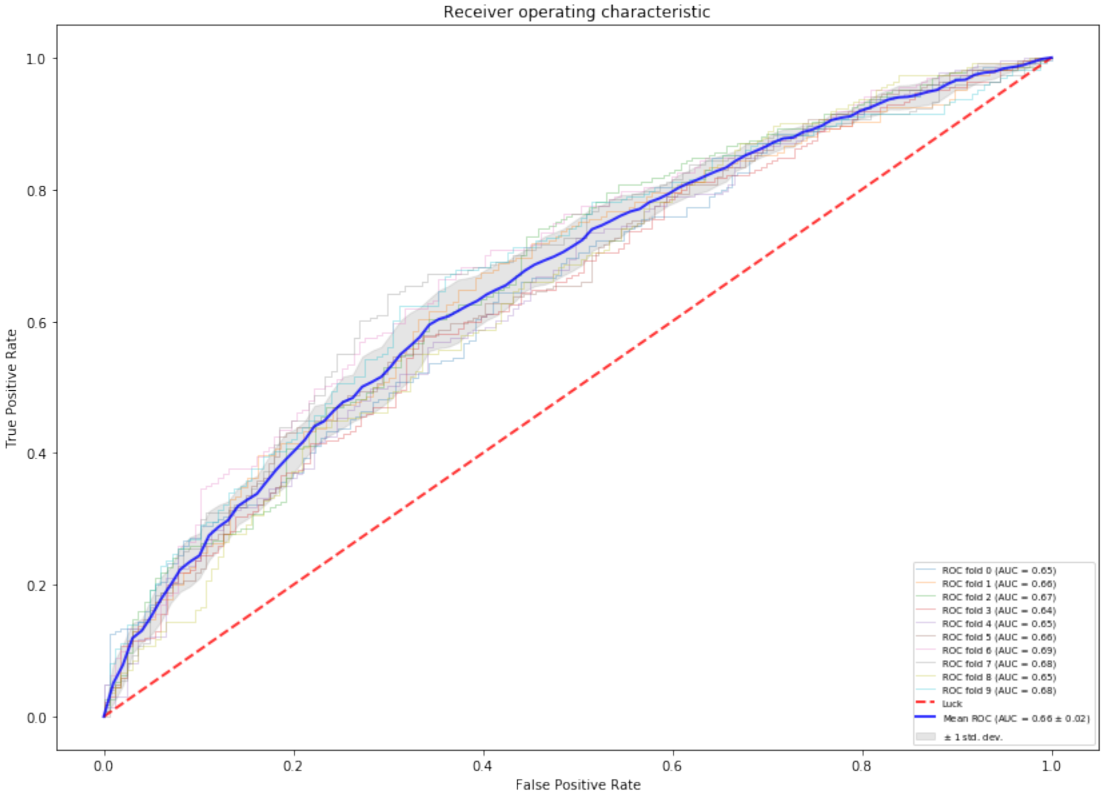

SDL Exclusive

Random forest:

Confusion Matrix

	T2D Negative	T2D Positive
T2D Negative	959	707
T2D Positive	404	1830

	Precision	Recall	F1-score
T2D Negative	0.70	0.58	0.63
T2D Positive	0.72	0.82	0.77

AUROC scores and ROC curve

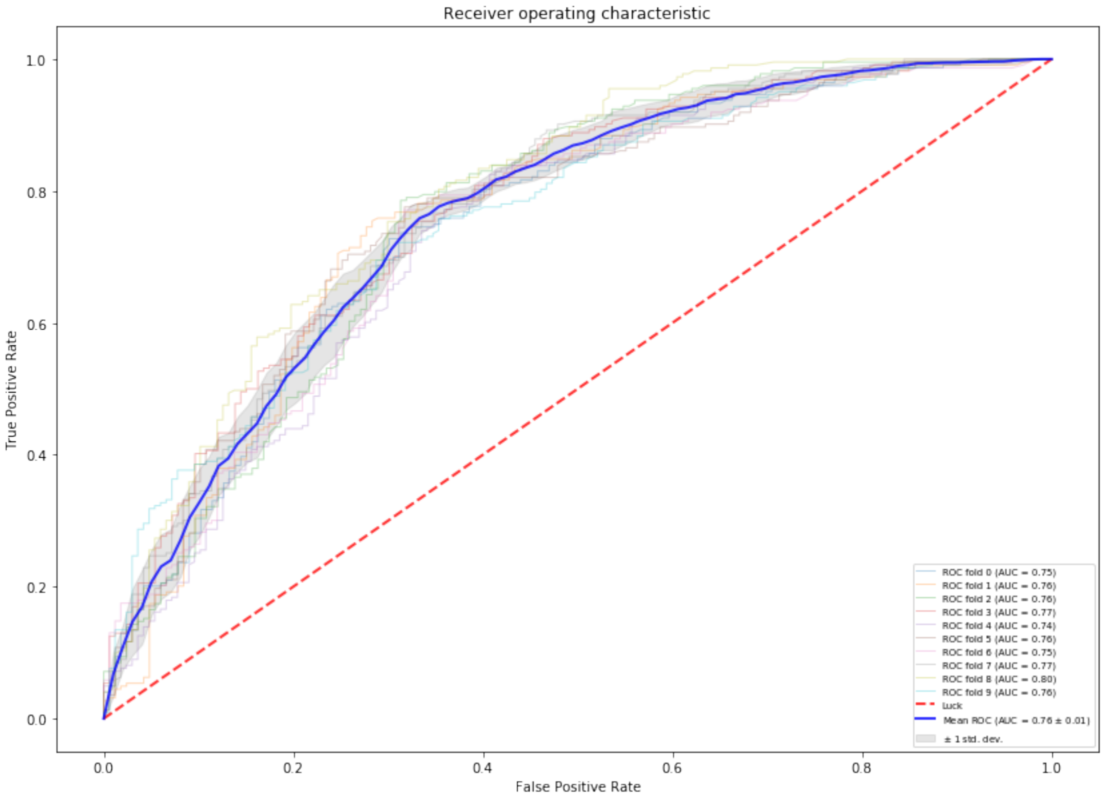

Logistic regression

Confusion Matrix

	T2D Negative	T2D Positive
T2D Negative	1016	650
T2D Positive	444	1790

	Precision	Recall	F1-score
T2D Negative	0.70	0.61	0.65
T2D Positive	0.73	0.80	0.77

AUROC scores and ROC curve

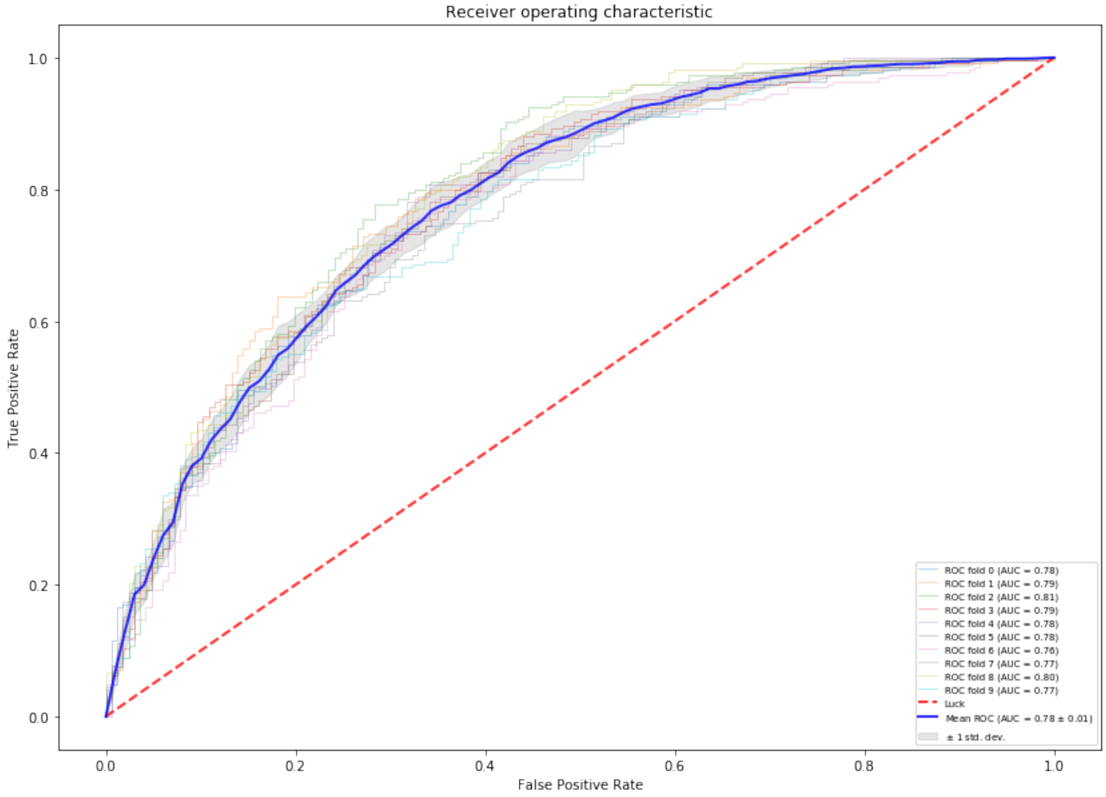

XGBoost

Confusion Matrix

	T2D Negative	T2D Positive
T2D Negative	962	704
T2D Positive	494	1740

	Precision	Recall	F1-score
T2D Negative	0.66	0.58	0.62
T2D Positive	0.71	0.78	0.74

AUROC scores and ROC curve

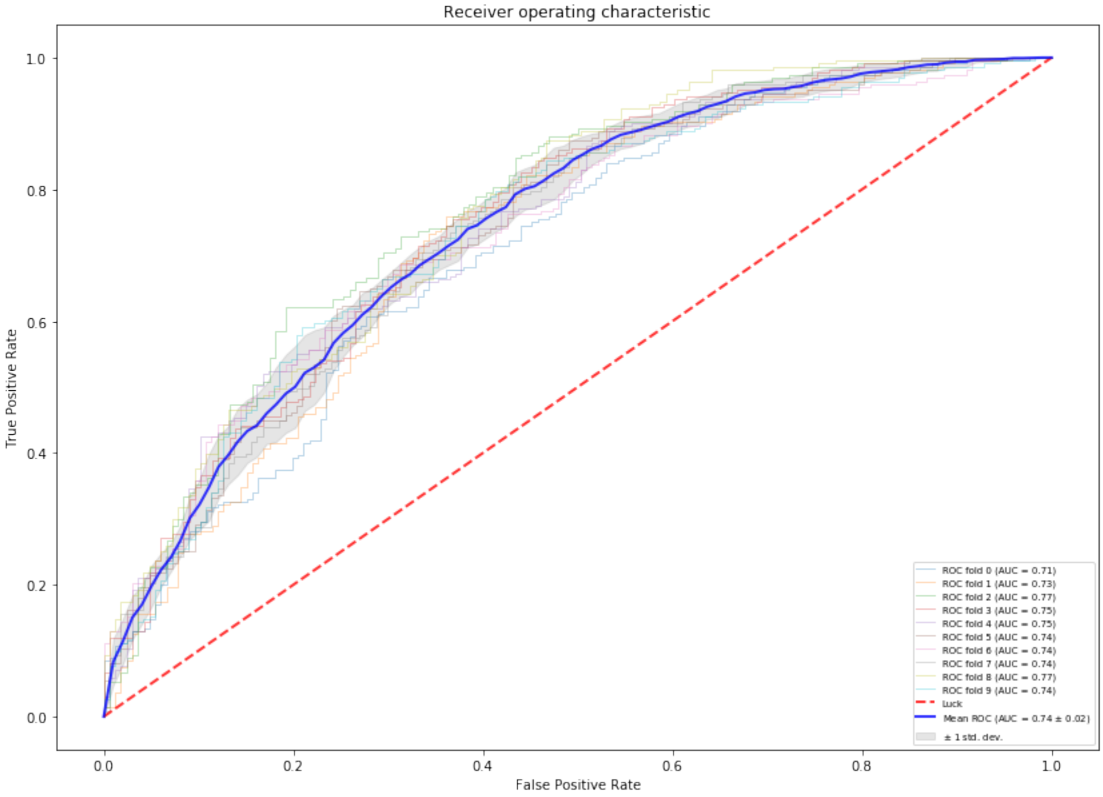

Supplement: Multimedia Appendix 3 [file diabetes_v6i1e23364_app3.pdf]
